# Genomic analysis of *Burkholderia* sp. ISTR5 for biofunneling of lignin-derived compounds

**DOI:** 10.1186/s13068-019-1606-5

**Published:** 2019-11-27

**Authors:** Raj Morya, Madan Kumar, Shashi Shekhar Singh, Indu Shekhar Thakur

**Affiliations:** School of Environmental Sciences, Jawaharlal Nehru University, New Delhi, 110067 India

**Keywords:** Lignin-derived compounds, Biodegradation, Lignin, Biofunneling, Genomics

## Abstract

**Background:**

Lignin is the second most abundant natural polymer on earth. Industries using lignocellulosic biomass as feedstock generate a considerable amount of lignin as a byproduct with minimal usage. For a sustainable biorefinery, the lignin must be utilized in improved ways. Lignin is recalcitrant to degradation due to the complex and heterogeneous structure. The depolymerization of lignin and its conversion into specific product stream are the major challenges associated with lignin valorization. The blend of oligomeric, dimeric and monomeric lignin-derived compounds (LDCs) generated during depolymerization can be utilized by microbes for production of bioproducts.

**Results:**

In the present study, a novel bacterium *Burkholderia* sp. strain ISTR5 (R5), a proteobacteria belonging to class betaproteobacteria, order *Burkholderiales* and family *Burkholderiaceae,* was isolated and characterized for the degradation of LDCs. R5 strain was cultured on 12 LDCs in mineral salt medium (MSM) supplemented with individual compounds such as syringic acid, *p*-coumaric acid, ferulic acid, vanillin, vanillic acid, guaiacol, 4-hydroxybenzoic acid, gallic acid, benzoic acid, syringaldehyde, veratryl alcohol and catechol. R5 was able to grow and utilize all the selected LDCs. The degradation of selected LDCs was monitored by bacterial growth, total organic carbon (TOC) removal and UV–Vis absorption spectra in scan mode. TOC reduction shown in the sample contains syringic acid 80.7%, ferulic acid 84.1%, *p*-coumaric acid 85.9% and benzoic acid 83.2%. In UV–Vis absorption spectral scan, most of the lignin-associated peaks were found at or near 280 nm wavelength in the obtained absorption spectra. Enzyme assay for the ligninolytic enzymes was also performed, and it was observed that lignin peroxidase and laccase were predominantly expressed. Furthermore, the GC–MS analysis of LDCs was performed to identify the degradation intermediates from these compounds. The genomic analysis showed the robustness of this strain and identified various candidate genes responsible for the degradation of aromatic or lignin derivatives, detoxification mechanism, oxidative stress response and fatty acid synthesis. The presence of peroxidases (13%), laccases (4%), monooxygenases (23%), dioxygenase (44%), NADPH: quinone oxidoreductases (16%) and many other related enzymes supported the degradation of LDCs.

**Conclusion:**

Numerous pathway intermediates were observed during experiment. Vanillin was found during growth on syringic acid, ferulic acid and *p*-coumaric acid. Some other intermediates like catechol, acetovanillone, syringaldehyde and 3,4-dihydroxybenzaldehyde from the recognized bacterial metabolic pathways existed during growth on the LDCs. The ortho- and meta cleavage pathway enzymes, such as the catechol-1,2-dioxygenase, protocatechuate 3,4-dioxygenase, catechol-2,3-dioxygenase and toluene-2,3-dioxygenase, were observed in the genome. In addition to the common aromatic degradation pathways, presence of the epoxyqueuosine reductase, 1,2-epoxyphenylacetyl-CoA isomerase in the genome advocates that this strain may follow the epoxy Coenzyme A thioester pathway for degradation.

## Background

Depleting fossil fuel reserves and climate change have compelled us to switch towards renewable alternatives for the production of fuels and chemicals. Lignocellulosic biomass is a renewable, easily available and low-cost alternative, which is being projected to replace Petro-based products. The lignocellulose biomass components include cellulose (40–50%), hemicellulose (20–30%) and lignin (10–25%) [1, 2]. Lignocellulosic-based industries utilize the biomass only after pretreatment to remove the lignin. There are various physical, chemical and biological methods developed for the pretreatment of biomass [3]. After pretreatment, the lignin components are generally burnt for fueling boilers or left unutilized in the waste streams. Due to rich aromatic content, lignin is a valuable waste from biomass industry [4]. Researchers worldwide are focusing on lignin and its components for conversion into value-added products (vanillin, bioplastics, biosurfactants, adipic acid, etc.) leading to minimal or zero waste lignocellulose-based industry. Utilization of waste for the production of value-added products will fulfill the concept of biorefinery [5, 6].

Lignin is a complex aromatic heteropolymer recalcitrant to degradation. The monomers present in lignin are linked by β-ether (45–55%), biphenyl (10–25%) and other types of linkages in varying amounts depending on the type and source of biomass [5]. Lignin is generally composed of three monomeric compounds: guaiacyl (G), sinapyl (S) and coniferyl (H). For efficient utilization of lignin as a substrate for valorization it must be depolymerized. After depolymerization of lignin, it breaks into a mixture of oligomeric, dimeric and monomeric LDCs. The lignin derivatives are toxic to most of the microbes and inhibit their activity during the treatment of wastewater [7]. The noxiousness of these LDCs is mostly due to the functional groups in the structure. Groups such as aldehydes and apolar substituents are highly toxic, and comparatively, carboxylic acid is less toxic [8].

Microbes while utilizing the recalcitrant compounds (PCBs, PAHs, trichloroethylene, lignin, etc.) as the sole source of carbon and energy undergo various types of stress. Due to adverse metabolic, respiratory pressure on the microbes, they adapt to cope with these types of stress for survival. They adjust to the harsh environment by modification in their extracellular secretory protein system and with the help of porin, peroxiredoxins, etc. Only a few species of the bacteria such as *Pseudomonas, Bacillus, Pandoraea and Burkholderia,* etc. have generally been reported to develop these types of modifications. Few strains of fungi *Humicola grisea* var *thermoidea, Trichoderma viride, Talaromyces bassochlamydoides,* etc. and bacteria *Bacillus* sp. LP1, *Pandoraea* sp. ISTKB, etc. were reported for the utilization of lignin and LDCs containing different functional groups [4, 9, 10]. Fungi have more potential to degrade lignin as compared to bacteria [11]. But there are advantages of using bacteria over fungi such as greater adaptability in diverse environments, and for further genetic manipulation due to the small genome size of bacteria, genetic modification becomes easy [11]. In nature lignin is mostly degraded by the action of extracellular enzymes such as laccases and peroxidases by different species of fungi. This action on lignin results in production of diverse array of low molecular weight aromatic compounds. Several microbes have evolved diverse catabolic pathways due to the existence of these aromatic molecules. There are different types of bacterial species which can utilize LDCs as a carbon source, e.g., *Sphingobacterium* sp., *Pseudomonas* sp., *Acinetobacter* sp. [3, 12]. Adetitun et al. showed the degradation of several LDCs like vanillin, vanillic acid, syringic acid, etc. by using gram-negative bacteria *Alcaligens* sp. 3K [13]. Nishimura et al. also displayed the utilization of LDCs such as ferulic acid, veratric acid, caffeic acid, protocatechuic acid, vanillic acid, coniferyl alcohol, isovanillic acid and vanillin as carbon sources by using *Streptomyces* sp. [14]. Several other researchers reported the degradation of lignin and LDCs by using the diverse bacterial strains like *Ralstonia*, *Sphingomonas, Bacillus,* *Klebsiella, Ochrobactrum tritici* [15, 16].

*Burkholderia,* a proteobacteria belonging to class betaproteobacteria, order *Burkholderiales* and family *Burkholderiaceae,* is widely known for the biodegradation of diverse types of substrates and other biotechnological applications [17–21]. Jung et al. used *Burkholderia glumae* BGR1 for the production of *p*-hydroxybenzoic acid from *p*-coumaric acid [18]. The *Burkholderia* sp. efficiently utilized indole, propiconazole, di-(2-ethylhexyl) phthalate (DEHP) [22, 23]. Use of this genus has also been shown to be consistent for degradation of organic pollutants, such as lindane, methyl parathion, polycyclic aromatic hydrocarbons, such as Benzo (a) pyrene, dibenz (a, h) anthracene, flouranthene, benzene (a), anthracene, etc. [24–26]. The report on LDC utilization by the *Burkholderia* genus is limited, thus limiting its application in lignin valorization.

There are limited reports available regarding the utilization of various LDCs by beta-proteobacteria. In the present study, a novel isolate R5 was able to metabolize a number of different aromatic compounds (syringic acid, ferulic acid, benzoic acid, *p*-coumaric acid, *p*-hydroxybenzoic acid, vanillin, vanillic acid, veratryl alcohol, guaiacol, catechol, gallic acid), which makes R5 a model strain for aromatic compound degradation studies. This strain was studied for the degradation of different LDCs containing different types of functional groups like aldehyde, carboxylic, methyl, methoxy and hydroxyl groups, etc. Enzyme assays (Lignin peroxidase (Lip), Manganese peroxidase (MnP) and laccase), total organic carbon, UV–Vis absorption spectral scan on spectrophotometer (200–800 nm wavelength) and GC–MS analysis were done to confirm the degradation of LDCs. The genomic analysis supported and highlighted the robust metabolic machinery of this strain. The findings will enhance the understanding of the role of *Burkholderia* genus in LDC utilization and its prospective application as a biofunneling bacteria for an economical biorefinery.

## Results and discussion

### Screening and characterization of bacterial isolates

**Fig. 1:**
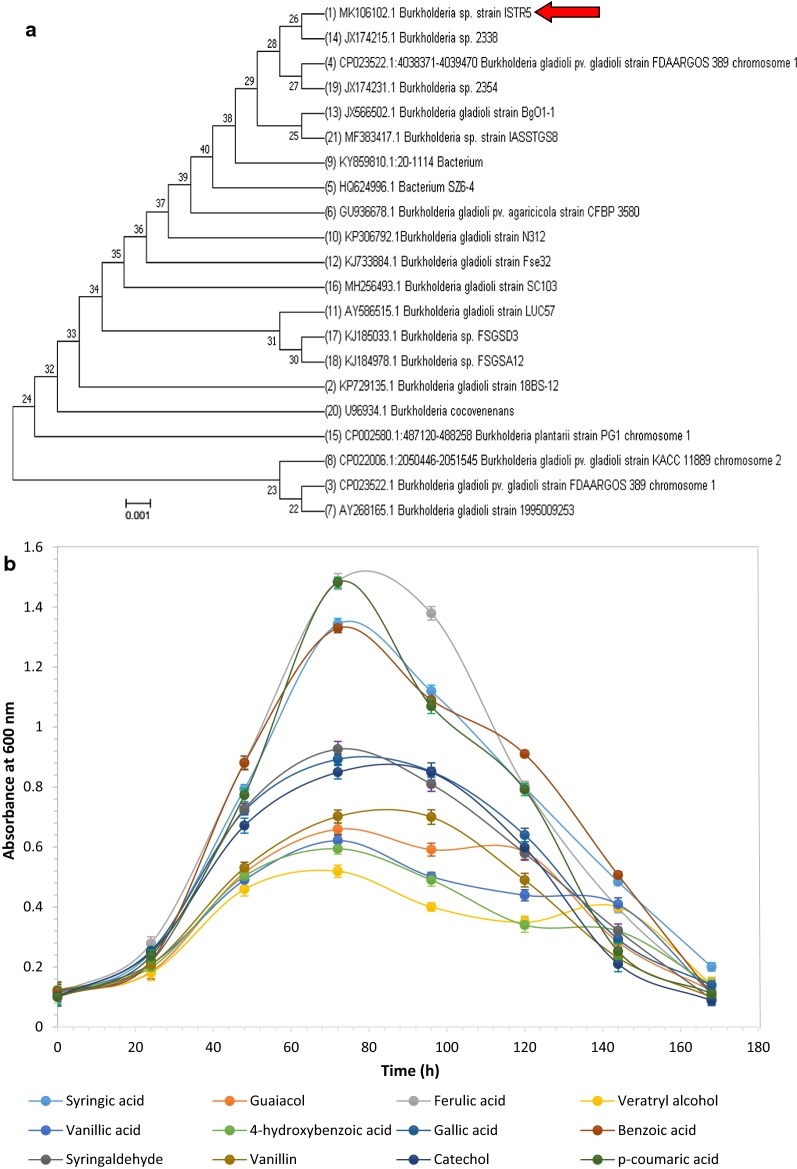
**a** Phylogenetic tree of *Burkholderia* sp. ISTR5 displaying maximum relationship based on 16S rDNA sequence. FASTA sequences were aligned using MUSCLE (MUltiple Sequence Comparison by Log- Expectation) and evolutionary history was inferred using the neighbor-joining method using the software MEGA 7. **b** Growth curve of *Burkholderia* sp. ISTR5 on different lignin-derived compounds (1 g/L) over 7 days of reaction at pH 8. Data shown as average value of three replicates and standard deviation as error bar (mean ± SD)

Morphologically different colonies of bacteria (R5, R6, R7, R8 and R9) were streaked on separate plates and were further screened for their potential for LDC degradation as sole carbon source. Although the degradation pattern was monitored for 168 h, the highest degradation was observed at 120 h for all the isolates. The bacterial strain R5 showed growth on all the LDCs used, followed by R8 with growth on 8, R7 and R6 on 6, R9 on 4 LDCs (Table 1). Due to its growth on maximum substrates, strain R5 was selected for further studies and was identified by 16S rDNA sequencing. 16S rDNA sequence was subjected to similarity search using NCBI BLAST; the result showed 98% identity to *Burkholderia* sp. considered as a novel strain. R5 has been submitted in the NCBI GenBank database with accession no. MK106102.1. R5 strain has 98% homology with *Burkholderia gladioli* (Fig. 1a). Till date, there are minimal reports available on the usage of LDCs by *Burkholderia* sp.

**Table 1:** Growth of different bacterial strains (R1, R2, R3, R4, and R5) on lignin-derived compounds used in the study for selection of potent bacteria for degradation

LDCs/strains	R5	R6	R7	R8	R9
Syringic acid	+	−	−	−	−
Guaiacol	+	+	+	+	−
Ferulic acid	+	+	−	+	+
Veratryl alcohol	+	−	−	−	−
Vanillic acid	+	−	+	+	+
4-Hydroxybenzoic acid	+	+	+	+	−
Gallic acid	+	−	−	+	−
Benzoic acid	+	+	+	+	+
Syringaldehyde	+	−	−	−	−
Vanillin	+	−	+	−	−
Catechol	+	+	+	+	+
*p*-Coumaric acid	+	+	−	+	−

Growth +, no growth −

R5 strain showed the growth on all LDCs, but few compounds such as ferulic acid, benzoic acid, syringic acid and *p*-coumaric acid were preferred substrate and showed higher growth on these compounds. R5 strain also showed considerable growth on syringaldehyde, gallic acid, catechol, guaiacol, veratryl alcohol, vanillin, vanillic acid and *p*-hydroxybenzoic acid (Fig. 1b). Utilization of diverse LDCs indicates the potency of this strain to degrade toxic LDCs as sole carbon source. Growth of *Burkholderia* sp. on LDCs has also been reported [17, 27]. But the ambiguity of these studies was the growth of the strain on a limited number of LDCs, resulting in the partial explanation of the pathways and enzymes involved for growth on LDCs encountered during lignin degradation.

### Reduction in total organic carbon (TOC)

**Fig. 2:**
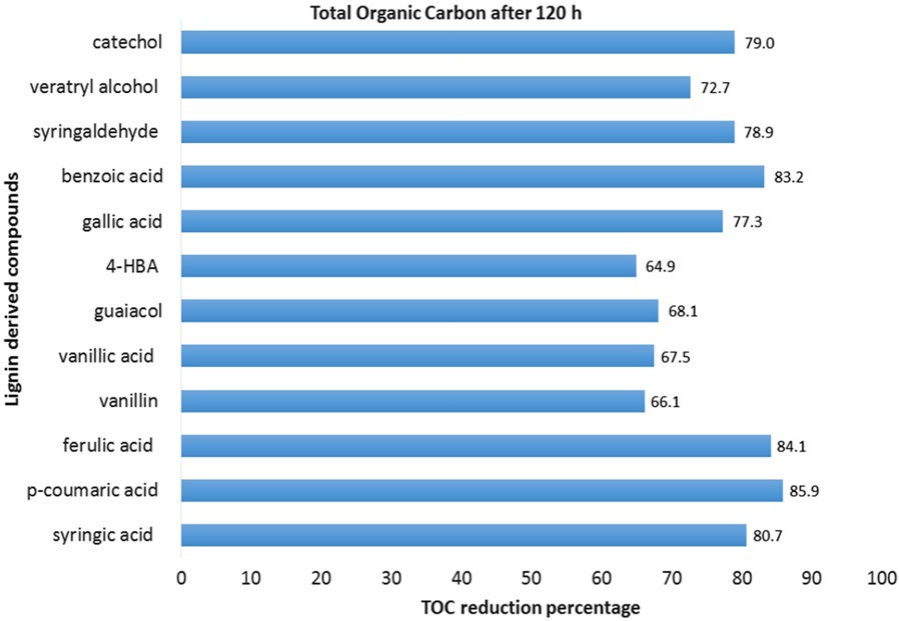
Total organic carbon reduction percentage of different LDCs when grown in MSM at concentration of 1 g/L at 30 °C measured after a time period of 120 h

LDCs were the only carbon source contributing the entire TOC of the reaction mixture. Therefore, a directly proportional relation of the TOC with LDC degradation was established. The findings asserted that strain R5 could mineralize the various types of LDCs used in the study. Decrease in percentage of TOC is as follows: syringic acid is 80.7%, *p*-coumaric acid 85.9%, ferulic acid 84.1%, vanillin 66.1%, vanillic acid 67.5%, guaiacol 68.1%, 4-HBA 64.9%, gallic acid 77.3%, benzoic acid 83.2%, syringaldehyde 78.9%, veratryl alcohol 72.7% and catechol 79% (Fig. 2). The *Sphingobacterium* sp. has also been reported to have TOC reduction up to 82%, 61% and 44% while using vanillic acid, *p*-coumaric acid and syringic acid, respectively, as sole substrate [11]. In comparison to the published report, the present study using R5 strain indicated excellent degradability of LDCs. It shows the optimistic future for biovalorization and biodegradation of LDCs.

### Bacterial enzyme assays for lignin degrading enzymes

**Fig. 3:**
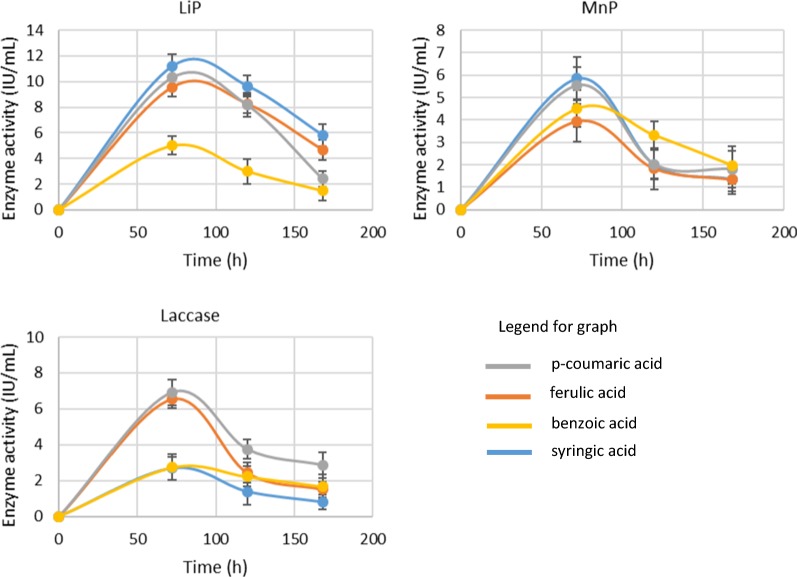
Enzyme activities of LiP, Laccase, and MnP in1 g/L LDCs-MSM for 7 days of incubation at pH 8 by *Burkholderia* sp. ISTR5. Experiments have been conducted in triplicate (mean ± SD)

R5, a gram-negative bacterium secretes various enzymes for the utilization of different types of aromatic substrates. Extracellular enzymes are a potent tool of microbes for complex aromatic compounds’ degradation [28]. Extracellular enzymes use oxidoreductive processes for the degradation of substrates. According to the earlier published research, there are three types of bacterial enzymes which are responsible for the degradation of lignin and LDCs are LiP, MnP and Laccase [29]. LiP, MnP, and Laccase activity is exhibited by the strain R5 during the growth on all the LDCs. On syringic acid, maximum LiP activity of 5.5 U/mL and Laccase activity of 4.8 U/mL were observed after 72 h. The result of the study indicated maximum activity of MnP (3.78 U/mL) at 72 h which was reduced to 2.9 and 2.5 U/mL at 96 and 120 h, respectively. Enzyme activity profile on *p*-coumaric acid was also quite interesting; MnP started showing the activity of 2.93 U/mL after 48 h and achieved maximum of 3.4 U/mL at 96 h. LiP started very slowly on this substrate with an activity of 0.9 U/mL and reached a maximum of 6.85 U/mL at 72 h. Laccase also showed the prominent maximum activity of 5.79 U/mL at 72 h. On ferulic acid, LiP is most active enzyme followed by MnP and Laccase (Fig. 3). Most dominating activity on benzoic acid was of laccase and MnP enzymes; LiP did not show activity in comparison to the two enzymes. Initially, low activity of all enzymes was observed on the substrates used. This could be due to the toxic effect of these LDCs for which bacteria required time to adapt to the culture conditions; activity increased with the adaptability of the bacteria. Enzyme activity data of syringic acid, *p*-coumaric acid, ferulic acid and benzoic acid supported the UV-Spectrophotometer scan mode analysis. Only these compounds were further studied for degradation. These enzymes are potent lignin degraders and been earlier reported by many researchers to degrade lignin and its related intermediates observed during biodegradation [30]. Wang et al. showed the maximum enzyme activity of Lip and MnP on syringic acid 17.5 U/L and 6 U/L, *p*-coumaric acid 13 U/L and 5 U/L, respectively [11]. These enzymes are potent lignin degraders and been earlier reported by many researchers to degrade lignin and its related intermediates observed during biodegradation [15, 30, 31].

### Degradation pattern analysis of lignin-derived compounds by UV–Vis spectral scan

**Fig. 4:**
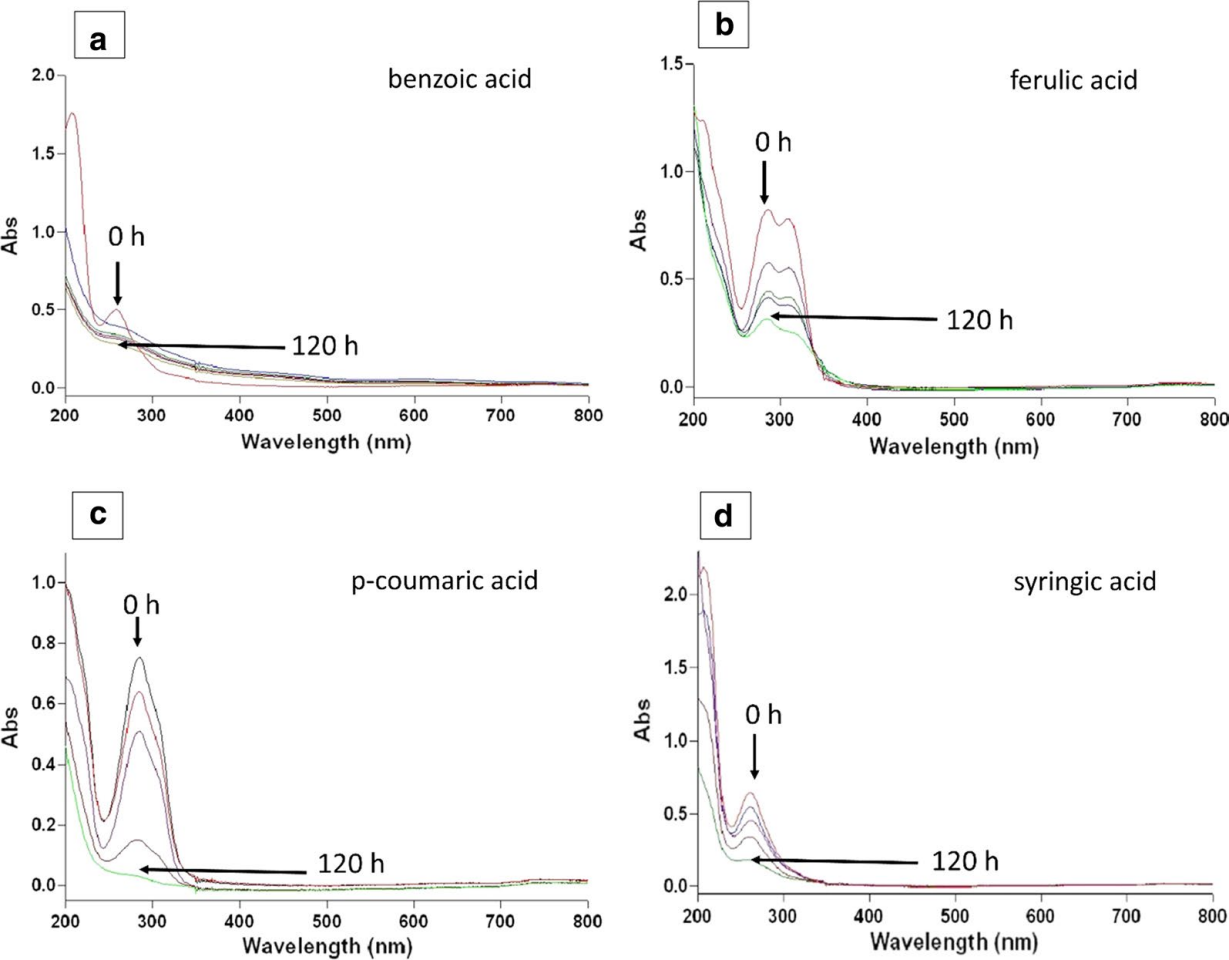
UV–Visible spectra of *p*-coumaric, syringic, ferulic, and benzoic acid grown in MSM. Comparison done from zero to 120 h

Preliminary studies for degradation of the various LDCs were assessed using scan mode. The aromatic compounds generally have signature peaks at or near 280 nm. In this study, the substrate concentration of 1 g/L was used. Degradation of the LDCs was visible with increasing time. The significant peak of the compounds at nearly 280 nm was completely diminished after 120 h of experiment. Few compounds which got significantly degraded are shown here in Fig. 4.

### Prediction of intermediate metabolites obtained using GC–MS

**Fig. 5:**
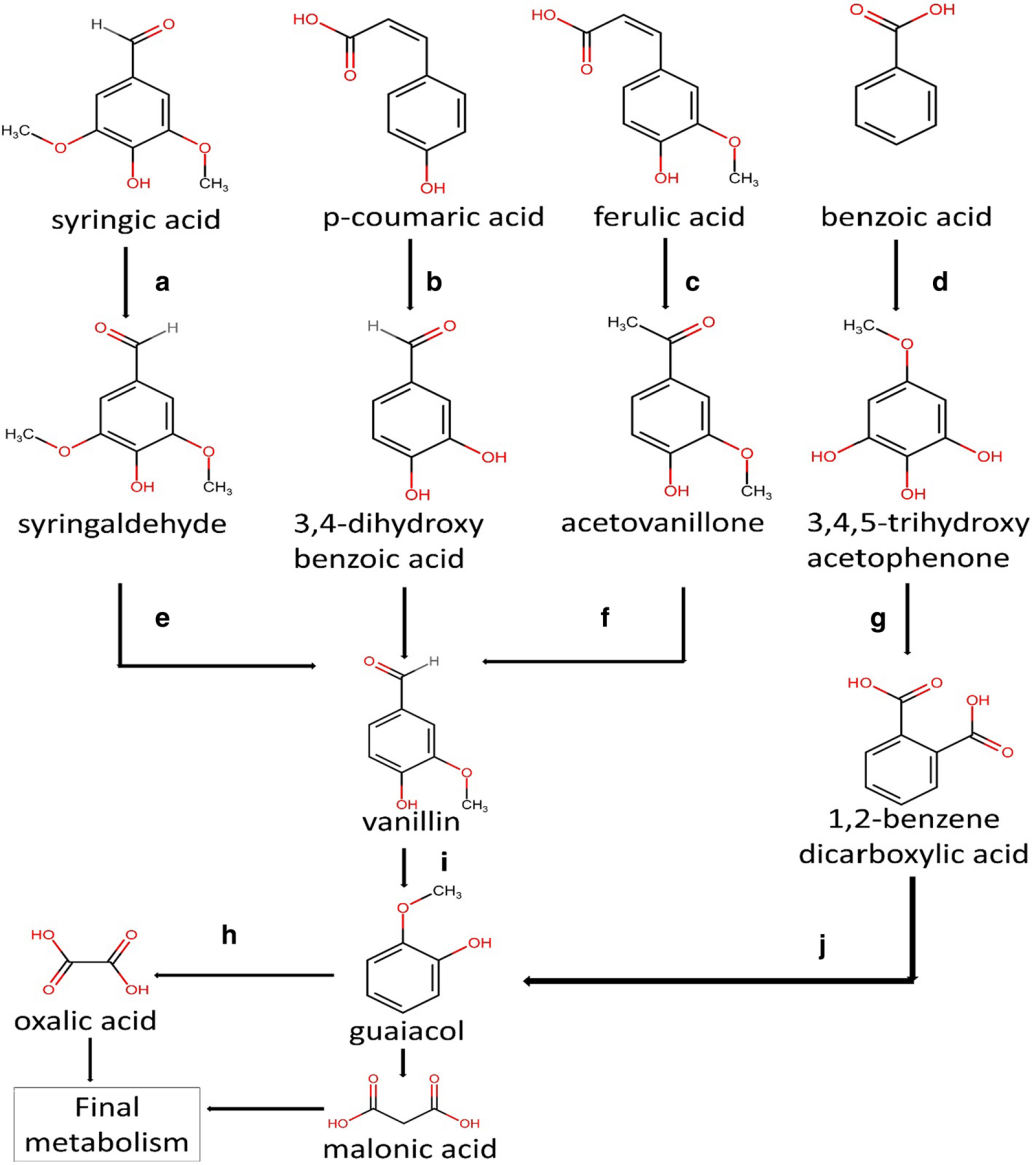
Degradation intermediates of lignin-derived compounds and their possible pathway prediction on the basis of GC–MS data. Enzyme responsible are a phenylacetate-CoA oxygenase reductase, b *p*-hydroxyphenylacetate 3-hydroxylase, c feruloyl-CoA synthetase, β-ketothiolase, d *p*-hydroxyphenylacetate hydroxylase, e enoyl-CoA aldolase, methyltransferase, f enoyl-CoA hydratase, g enoyl-CoA aldolase, h *O*-demethylase, catechol 1,2-dioxygenase, i vanillin dehydrogenase and j laccase. Chemical structures were drawn using https://chem-space.com/search

GC–MS was done to analyze the sample qualitatively and to identify the intermediates produced during the degradation of the substrate. The GC–MS analysis was performed to explain the degradation mechanism of the LDCs. The number of peaks in the chromatogram showed the number of compounds present in the sample (Additional file 1: Figure S6). With increasing reaction time, the number of peaks in the chromatogram increased due to the production of a varying number of metabolites of LDCs. These produced fragments were found to follow different pathways for the final metabolism of LDCs. Here only four LDCs (showing highest growth) were selected for the degradation and identification of metabolites, and their degradation pathway is mapped in Fig. 5.

Syringic acid belongs to the lignin structure’s sinapyl monomer, which contains two methoxy, one hydroxyl and one carboxylic functional groups. The 72-h degradation sample spectra showed peaks of vanillin (RT 8.5), syringaldehyde (RT 13.5) and isovanillin (RT 9.3). Essential enzymes which are responsible for conversion of syringic acid into vanillin include phenylacetate-CoA oxygenase reductase, Enoyl-CoA aldolase and methyltransferases. [32]. 120 h sample includes peaks of vanillin (RT 8.13), oxalic acid (RT 22.9), guaifenesin (RT 13.4) and guaiacol (RT 13.5). Syringic acid degraded completely via ortho cleavage pathway as evident from the presence of guaiacol and oxalic acid.

*p*-Coumaric acid representing coumaryl monomer of lignin structure is mostly found in the grasses [33]. 3,4-dihydroxybenzaldehyde (RT 9.6) and vanillin (RT 8.2) were the key intermediates found after 72 h of reaction that may be due to the reduction of *p*-coumaric acid using *p*-hydroxyphenylacetate 3-hydroxylase, reductase component. Following the oxidation, reduction reaction to the final products after 120 h sample mainly comprised guaiacol (RT 13.6) and malonic acid (RT 21.4). From the intermediates obtained, it is evident that the degradation pattern of *p*-coumaric acid is similar to the β-ketoadipate pathway.

Ferulic acid represents the coniferyl monomer of the lignin structure. Degradation profile indicated that the main metabolite present after 72 h of incubation was vanillin (RT 8.3), acetovanillone (RT 9.8) and in trace amounts, vanillic acidand catechol; similar results were reported by [34]. After 120 h, the main metabolite extant was guaifenesin (RT 13.5) and vanillin (RT 8.2). Aldehydic group shows negative effects on the growth of bacteria that could be the reason that the vanillin was not utilized within this period [35].

Benzoic acid is a representative of benzoyl monomer of the lignin structure; its degradation occurred through the β-ketoadipate pathway. After 72 h of reaction, peaks of intermediates like 3, 4, 5-trihydroxyacetophenone (RT 9.8), 1,2-benzenedicarboxylic acid (RT 29.4) were found in the spectra. 120-h sample contains peaks of maleic acid (RT 34.5), Oxalic acid (RT 11.9) and muconate (RT 13.5). The product profiling of the benzoic acid degradation signals towards the degradation via beta-ketoadipate pathway; similar pathway was also hypothesized by other researchers [11, 36].

The common intermediate obtained in the degradation of all the LDCs was vanillin. This shows the importance of biological funneling, i.e. bacteria can effectively degrade the various LDCs and bring at a common product. This funneling of the compounds paves the way for lignin valorization study.

### *Burkholderia* sp. ISTR5 genome analysis for lignin-derived compounds’ degradation

R5 proved to be efficient for the degradation of LDCs. The genome size of this strain is found to be 8.16 Mb with GC content of 68.11, and other general genome features are given in (Table 2). KEGG-KAAS analysis distributes the number of genes according to their functions: 2393 protein coding genes were categorized into 22 KAAS pathway (Additional file 1: Table S1). EGGNog pathway analysis revealed that 114 proteins were responsible for the utilization and degradation of xenobiotic and aromatic compounds.

**Table 2:** General genome features of *Burkholderia* sp. ISTR5

Total bases (genome)	8,160,457 bp
Total proteins	6886
Total number of scaffolds	36
Scaffold N50	572,579 bp
Maximum scaffold size	1,042,056 bp
Minimum scaffold size	413 bp
GC percentage	68.11
Coverage percentage	65
Total sequence length	8.1 Mb
Total number of genes	7000
Pfam-annotated genes	2859
SignalP-passed proteins	764
Hypothetical proteins	983
rRNAs	4
tRNAs	77
tmRNA	1

### Respiratory mechanism, transcriptional factors and transporters in *Burkholderia* sp. ISTR5 genome

Different electron donors, electron acceptors and other pertinent genes attributed to respiration are present in the genome. Surplus quinone oxidoreductases, soluble cytochromes, formate dehydrogenases and additional related electron transporters illustrate the significance and support in the metabolism of recalcitrant compounds (Additional file 1: Figure S1). Formate dehydrogenases, ubiquinol oxidases, oxidoreductases, quinone oxidoreductases family proteins and several electron carriers’ abundance highlight the potency of this strain to sustain under toxic environment and utilize them to derive food and energy [4]. Various enzymes needed for cellular respiration were present in the genome of R5. Bacterial respiration takes place via respiratory chain mechanism consisting of (i) NADH-quinone oxidoreductase, (ii) succinate dehydrogenase, (iii) cytochrome c oxidoreductase and (iv) cytochrome c oxidase. In addition to it, there were many other enzymes such as ubiquinone methyltransferase, quinone oxidoreductase and cytochrome ubiquinol oxidase also present.

Genomic analysis showed the presence of 518 transcriptional factors; from this, nearly 46% of the regulators belong to the LysR family; several other regulators like GntR, IclR, XRE and MarR families were also present, which showed the robustness for efficient utilization of aromatic hydrocarbons [37]. Presence of CatM and BenM in the genome indicates the robustness of bacteria for efficient aromatic hydrocarbon degradation [17]. BenM and CatM are involved in the regulation of operons, which encodes the genes for the degradation of benzoate/catechol to *cis*,*cis*-muconate [4, 6]. CatM regulates the benPK and catBCIJFD operons responsible for the *cis*,*cis*-muconate to the tricarboxylic acid cycle. PcaQ regulates the operons which are involved in the conversion of protocatechuate into β-Carboxy-*cis*,*cis*-muconate and γ-carboxymuconate; these products were observed in the GC–MS analysis of the substrates experimented for the degradation [37]. Generally, LysR family regulators are related to ortho-cleavage pathway of catechol, but other pathways are also possible. IclR family regulator PcaR controls the ortho-cleavage pathway enzymes and MhpR controls the operons for meta-cleavage of an aromatic compound. Likewise, MarR found in the genome is responsible for controlling the metabolism and response of the bacteria towards the aromatic substrates [38]. Similarly, hydroxycinnamic acid derivatives such as ferulic acid and *p*-coumaric acid tested in the study may be degraded by strain R5 due to the presence of gene HcaR [39]. Transporters are required for the movement of ions, proteins and other molecules through a cellular membrane which is essential for the biodegradation of substrates. A total of 527 transporters are found in the strain, out of which ABC transporter superfamily accounts for the 45% of total transporters, followed by MFS (~ 20%) and RND (5%). The genome of the bacteria contains several transporters essential for the transport and degradation of the aromatic substrates. ABC transporters are known for their role in efficient transport of lignin derivatives such as vanillin, benzoate. Most common transport regulators involved in lignin are ABC, MFS, TRAP and member of Ion transport superfamily [37]. ABC transport system is highly substrate-specific, and solute binding protein-based studies reported the identification of LDCs like sinapyl, coumaryl and coniferyl. The MFS transporters are largely involved in the protocatechuate, ferulate and benzoate transportation. TRAP and IT were also shown to be active for the transportation of LDCs [40].

### Metabolism of lignin-derived compounds

Annotation of R5 genes and further their classification into pathways involved in the degradation of lignin or aromatic compounds have been identified by KEGG pathway analysis, blast search against the ‘nr’ database and RAST subsystem feature. 25 monooxygenases, 48 dioxygenases, 15 peroxidases including two DyP-type peroxidases, 17 quinone oxidoreductases were found in the COG analysis (Additional file 1: Tables S2–S4, Figure S4). Different oxidoreductases (grouped into flavin, FAD, pyridine nucleotide-disulfide, NAD(P)H, YggW, SDR, Fe–S, GMC, quinone and non-classified oxidoreductases), hydrolases, reductases, thio-esterases, dehydrogenases, transferases and esterases were also observed.

The pathway studies suggest that genes responsible for the degradation of lignin and various aromatic compounds were present in the genome (Fig. 5). Genes responsible for the degradation of lignin or aromatic components have been observed via peripheral degradation pathways. Genes related to phenylpropanoic acid, vanillin, biphenyl, ferulate, mediated by phenylacetate, benzoyl-CoA and phenol degradation have been found (Additional file 1: Table S5). Lignin fragment degradation genes which are responsible for the metabolism of lignin and LDCs were also found in the subsystem features. The KEGG investigation shows that this strain can use various xenobiotic compounds such as aminobenzoate, ethylbenzoate, *p*-hydroxybenzoate, benzofluoride, PAH, salicylate esters, pesticides, BTEX, furfurals, quinates and steroids. Generally, central metabolic pathways, beta-ketoadipate pathway and aromatic ring cleaving pathways are responsible for the final metabolism of aromatic compounds. Genes responsible for the transformation of central intermediates into final metabolism were observed. Enzymatic genes present in the bacteria are found to be coherent with the literature for the degradation of aromatic compounds via ortho and meta-cleavage pathways [41].

### Stress response mechanism-related genes

The known enzymes for lignin degradation are the oxidoreductases such as LiP, MnP, Laccase and other accessory enzymes. On reaction with the aromatic compounds, they generate reactive intermediates called free radicals. Free radicals are toxic for the bacteria and affect the DNA and proteins. To survive in this environment, bacteria have to develop genetic machinery to modify these free radicals into stable form or to neutralize it by using several enzymes such as SOD (superoxide dismutase), glutathione transferase, catalase, etc. [28]. These types of enzymes play a significant role in the survival of the bacteria. An array of enzymes exhibiting detoxification and stress response mechanism was also present in the genome (Additional file 1: Figure S5 and Table S7). The occurrence of catalases, thioredoxin, superoxide dismutase, aldo/keto reductase, alkyl hydroperoxidase, glyoxylase, peroxiredoxin, etc. showed the strong collection of enzymes to cope with the oxidative stress produced due to the actions of oxidoreductases [42].

### Lignin-derived compounds’ degradation machinery analysis of *Burkholderia* sp. ISTR5 based on whole genome sequencing

*Burkholderia* genus is known for its aerobic mode degradation of aromatic hydrocarbons (aromatics generally degrade by aerobic mode). Numerous genes related to respiration, stress response and aromatic metabolism are present in the genome of strain R5. Transporters, receptors, metal ion binding and other membrane proteins found in the genome showed the presence of active transportation and metabolism machinery for the utilization of substrates.

The genomic profile showed the presence of various genes encoding enzymes, which are essential for the scavenging of harmful chemical species in the culture media. Alkyl hydroperoxide reductase is responsible for the reduction of H_2_O_2_ and organic hydroperoxides produced into water and alcohols, respectively. Detoxification of peroxides by alkyl hydroperoxide reductase plays a vital role in cell protection from oxidative stress [43]. Apart from stress management in the cell, various glutathione transferases, dehydrogenases and hydrolases were found; earlier studies reported glutathione-s-transferase for the degradation of lignin-related compound [44]. Thus, the presence of glutathione can aid in the degradation of LDCs in addition to overcome the stress.

During the degradation process of aromatics by an oxidoreductase, free radicals are generated. One of them is superoxide O^2−^, which is converted into O_2_ or H_2_O_2_ by Superoxide dismutase (SOD); this enzyme helps to cope up with the oxidative stress. Different types of SODs were found in the genome and have been recently shown to be responsible for demethylation of lignin [45]. Kumar et al. showed the presence of various demethylases, SAM-dependent methyltransferases and other methyltransferases plays an essential role in the rearrangements of methyl groups [46]. In the present study, LDCs such as syringic acid, ferulic acid, *p*-coumaric acid and other substrate’s degradation were hypothesized to proceed through demethylation and methyl shift reaction. Methoxy functional group-containing compounds like syringaldehyde, vanillin, and guaiacol were degraded by strain R5 due to the presence of Rieske type Fe–S cluster in demethylases enzyme [47]. In addition, the substrate containing aldehydic functional group indicated considerable degradation in the present study. Dehydrogenase enzyme family degrades toxic aldehydes and transforms them into less harmful compounds. Dehydrogenases associated with aromatic aldehydes were observed in the strain, such as aldehyde dehydrogenase, NAD-dependent succinate semialdehyde dehydrogenase, acyl-CoA dehydrogenase, alcohol dehydrogenase, quinate dehydrogenase, 4-formylbenzenesulfonate dehydrogenase, etc. [48]. Benzoate 1,2-dioxygenase is responsible for the conversion of benzoic acid into 4-hydroxyphenylacetate. Degradative proteins like arylesterase, NAD-dependent succinate-semialdehyde dehydrogenase, GMC oxidoreductase and 4-hydroxyphenylacetate 3-monooxygenase have also been found in the genome indicating the potency to break the basic skeleton of lignin [49, 50]. NADH-quinone oxidoreductase undergoes Fenton reaction, to donate an electron from NADH to quinone-like molecules (LDCs are structurally similar to quinone) [51]. Oxidases play a vital role in the functioning of the bacteria, catalyze the redox reaction and use oxygen as an electron acceptor. Various peroxidases use FAD-linked oxidase, cytochrome c oxidase and H_2_O_2_ generated by them for dynamic participation in LDCs degradation [49].

Lip, MnP and laccase enzyme activity shown by the strain R5 on different LDCs showed the presence of peroxidase and laccase in the genome. Catalase/peroxidase is a potential catalase enzyme which performs the function of peroxidase and is responsible for the degradation of aromatic hydrocarbons. Several peroxidase and multi-copper oxidases were observed in the genome, reiterating its robustness for the degradation of LDCs. In addition to Lip, MnP, and laccase enzymes, other key enzymes for lignin degradation such as enoyl CoA hydratase, acetyl-CoA hydratase, dehydrogenases, and peroxidases are also present in the genome of strain R5 which is similar to *Bacillus ligniniphilus* L1 [52].

Genomic profile of R5 shows the existence of different pathways; one of them is Epoxy–CoA facilitated degradation of aromatic hydrocarbons. Epoxy CoA-thioester pathway genes were found in the genome of the bacteria. The aromatic compounds are degraded by the formation of epoxide bond by enzymes Acyl CoA reductase and 1,2-phenylacetyl CoA epoxidase. The enoyl CoA hydratase and Epoxide hydrolase are responsible for the degradation of intermediate and formation of diol derivative, respectively. Further metabolism to final products takes place using NAD-dependent succinate semialdehyde dehydrogenase [53]. This type of degradation mechanism is sporadic in aerobic bacteria; only a few reports reported to follow this extraordinary pathway like *Pseudomonas putida, Escherichia coli* and *Pandoraea* sp. [45]. The genomic profile showed the presence of enzymes Protocatechuate 3,4-dioxygenase, Protocatechuate 4,5-dioxygenase known for ortho and meta cleavage of aromatic compounds, respectively [47]. Presence of both the ring cleavage pathway makes this bacteria a strong candidate for LDCs degradation. Most of the LDCs used were degraded either through a meta or ortho cleavage pathway. Some acyl CoA transferases, kinases, hydrolases, dehydrogenases, reductases and synthases were observed in the genomic profile. These enzymes are responsible for the decarboxylation reaction and divert the degradation towards the central pathway [54].

The analysis and level of existence of such a diverse gene set have helped us identify key lignin or aromatic biodegradation enzymes which will also allow for the valorization of lignocellulosic biomass.

## Conclusion

We have isolated and characterized a bacterial strain from the soil, capable of utilizing various types of LDCs. Based on experiments performed and data analysis, we infer the remarkable inherent capabilities of the strain R5 for the utilization of 12 LDCs. Strain R5 identified as *Burkholderia* sp. based on the 16S rDNA sequencing analysis, possesses most of the key upper funneling pathways required for the transformation of LDCs. In addition to dominating enzymes such as Laccase and peroxidase, it has genomic machinery that plays an important role in the protection and survival of the cells against the harsh environment and oxidative stress. Along with the beta-ketoadipate pathway, this strain also has machinery for the Epoxy CoA-thioester pathway for the degradation of substrates. Conclusively, the study analyzed the core genes present in the genome of R5 for LDC utilization and represented the metabolic pathways followed for the degradation of LDCs. The intermediate metabolic compounds formed during degradation of syringic, ferulic, and *p*-coumaric acid indicated vanillin mediated degradation of the compounds. Biofunneling of complex aromatic compounds generated from lignin overcomes the heterogeneity and provides opportunity for bioconversion.

## Materials and methods

### Chemicals and reagents used in the present study

All the chemicals used, syringic acid (C_9_H_10_O_5_), *p*-coumaric acid (C_9_H_8_O_3_), vanillic acid (C_8_H_8_O_4_), ferulic acid (C_10_H_10_O_4_), vanillin (C_8_H_8_O_3_), guaiacol (C_7_H_8_O_2_), 4-hydroxybenzoic acid (C_7_H_6_O_3_), gallic acid (C_7_H_6_O_5_), benzoic acid (C_7_H_6_O_2_), syringaldehyde (C_9_H_10_O_4_), veratryl alcohol (C_9_H_12_O_3_) and catechol, (C_6_H_6_O_2_) in the present study were procured from the Sigma Aldrich (United States), Hi-Media (Mumbai, India), Merck and Tokyo Chemical Industry co. Ltd. (Japan) unless and otherwise stated.

### Sampling, isolation and screening of bacterial strain for LDCs degradation

Soil samples were collected from the Jawaharlal Nehru University, New Delhi (28.5402° N, 77.1662° E) forest soil where the degradation of wood was taking place. 1 g soil was mixed with 50 mL autoclaved distilled water in a sterile falcon tube and vortexed for proper mixing; 1 mL solution taken from it and transferred into 250 mL conical flask containing 50 mL 1 g/L Kraft lignin-MSM and cycloheximide (50 mg/L) was used to inhibit any fungal growth. The composition of Mineral Salt Media (MSM) used (g/L) was 7.8Na_2_HPO_4_·2H_2_O; 6.8KH_2_PO_4_; 0.04MnSO_4_; 0.2MgSO_4_; 0.03CuSO_4_; 0.01NH_4_ (CH3COO)_3_Fe; 0.05Ca(NO_3_)_2_·4H_2_O; 0.085NaNO_3_ and 1 g/L Kraft lignin at 30 °C; rpm 180, pH 8 [55]. MSM was sterilized by autoclaving and substrates were added to cold MSM by using 0.25 µm syringe filter. After 30 days, 100 µL of the culture was plated on nutrient agar plate for isolation of lignin-consuming bacteria. Five different types of bacterial colonies appeared and were further streaked on single nutrient agar plates and randomlytitledasR5, R6, R7, R8 and R9. Subsequently, each one of them was inoculated into LDCs (1 g/L)-MSM syringic acid, *p*-coumaric acid, vanillic acid, ferulic acid, vanillin, guaiacol, 4-hydroxybenzoic acid, gallic acid, benzoic acid, syringaldehyde, veratryl alcohol and catechol to obtain a final OD of 0.1, to isolate and screen the potential strain. Conical flasks containing the stipulated reaction mixture were incubated at 180 rpm at 30 °C and pH8 was set using 0.1 N HCl and 0.1 M NaOH. For tracking the degradation trend of these LDCs, 2 mL sample was taken out daily for bacterial growth for 168 h.

### Characterization of potential isolate and phylogenetic analysis

The potential LDC degrading strain was identified using 16S rDNA sequence analysis. Bacteria were grown in 10 mL LB broth and the cell pellet harvested during exponential phase by centrifuging at 12,000 rpm for 5 min. Then, DNeasy Ultraclean microbial kit was used to extract the genomic DNA, as per manufacturer’s protocol. Amplification of 16S rDNA was carried out using primers P0 5′ (GAGAGTTTGATCCTGGCTCAG) 3′ and P6 5′ (CTACGGCTACCTTGTTACGA) 3′. Obtained 16S rDNA was gel purified using gel purification kit (Qiagen) and then sent for sequencing to Central Instrumentation Facility, University of Delhi South Campus. The Strain was identified after blast search of sequence in NCBI database and then phylogenetic tree was constructed using neighbor-joining method [30].

### Growth curve and TOC reduction of LDCs by novel isolate

Samples were withdrawn daily at a fixed time and absorbance was recorded followed by spectral scan using Varian Carry 100 Bio UV–Vis Spectrophotometer. Preliminary analysis of the degradation of LDCs was done using Varian Carry 100 Bio UV–Vis Spectrophotometer in the spectral scan mode [56]. In a pre-weighed falcon tube, bacterial culture media was centrifuged and the supernatant was kept at 4 °C for enzyme assay [30]. In a 50-mL beaker, 20 mL of the supernatant of the culture obtained after centrifugation was taken and acidified using 1 N HCl. After acidification, solution was filtered through a 0.25-µm syringe filter and then analyzed for TOC using Shimadzu TOC-L analyzer.

### Enzyme assay for lignin degrading enzymes

For spectrophotometric enzyme assay, the samples were centrifuged at 8000 rpm for 15 min and the supernatant was kept at 4 °C for Lignin peroxidase (Lip), laccase and Manganese peroxidase (MnP) enzyme assays. Laccase enzyme activity was analyzed by using ABTS as substrate. The reaction mixture comprises ABTS (0.5 mM) as substrate, enzyme, and sodium tartarate buffer (100 mM, pH 3) incubated for 10 min and then absorbance reading was determined at 420 nm at a time interval of 30 s for 3 min, ε420 = 36,000 M^−1^ cm^−1^ [57]. LiP assay was carried out on the basis of oxidation of Azure B by peroxidase enzyme. The reaction mixture contains sodium tartarate buffer (100 mM, pH 3), Azure B (0.125 mM), enzyme, and H_2_O_2_ (0.1 mM) [58]. LiP activity was measured by taking absorbance at 651 nm, ε651 = 48.8 M^−1^ cm^−1^ [58]. The manganese peroxide enzyme activity was determined by the change in absorbance where the Mn^2+^ oxidize to Mn^3+^ and formed complex with malonate which gives absorbance at 270 nm, ε270 = 11,590 M^−1^ cm^−1^ [30]. The reaction mixture constituents are 0.5 mL malonate buffer 50 mM (0.1 M, pH 4.5, 10.406 g/L malonic acid in water), 0.2 mL MnSO_4_ 1 mM (2.5 mM, 0.423 g/L MnSO_4_·H_2_O in water), 0.1 mL enzyme, 0.2 mL H_2_O_2_ 0.1 mM (0.5 mM in water). Enzyme activity is generally expressed in U/mL, defined as the volume of enzyme required to oxidize 1 μmol of the substrate per minute at 25 °C [30].

### Sample preparation for GC–MS

For GC–MS analysis, 50 mL sample withdrawn was centrifuged at 8000 rpm for 10 min and then acidified to pH 2–3. Then extraction was done using an equal volume of ethyl acetate, shaken for 30 min for proper mixing so that migration of organic compounds takes place in ethyl acetate. Thereafter, this solution was kept in separating funnel, and an organic layer containing LDCs was taken and dried to 2 mL; then 50 µL Pyridine and 100 µL BSTFA (*N,O*-bis(trimethylsilyl)trifluoroacetamide) were added for derivatization [52]. This solution was then nitrogen dried at the rate of 20 mL/s, 10 bar pressure for 25–30 min; again 1–2 mL of the suitable solvent (here ethyl acetate) was added and the samples were sent for GC–MS analysis. Shimadzu GC–MS–QP 2010 Plus fitted with Rtx-5MS capillary column (30 m-long, 0.25 μm-thick film, 0.25 mm ID) was used for the analysis. 1 µL of the derivatized sample was injected into injector port and operating conditions were as follows: starting temperature 60 °C for 3-min duration, hold time of 8 min and stepwise increment of 12 °C/min was set with initial temperature 60 to final 320 °C. Data obtained were compared with the standard mass spectral libraries such as NIST-17, Wiley-8 and Wiley-14 [59].

### Genomic analysis of R5 for the degradation of LDCs

Bacteria were inoculated in LB-broth and allowed to grow at 180 rpm, 30 °C for 14–16 h. Culture was harvested during log phase, centrifuged at 8000 rpm, at 4°C and supernatant was discarded. Bacterial pellet was used for DNA isolation for whole genome sequencing. Genomic DNA of the strain was isolated by using DNeasy Ultraclean microbial kit, Qiagen (Germany). The Illumina MiSeq platform was used for sequencing of R5 genome, and raw data processing, selection of quality reads, assembly, generation of scaffolds, and gene prediction were performed as described earlier [41]. Arrangement of the R5 genes on its genome was carried out via clicO FS [60]. Identification of signal proteins in the sequence has been done by SignalP 3.0 software. Rapid annotations using subsystem technology (RAST) were also used to annotate and analyze the R5 genome [61]. For identifying the genes involved in biological pathways, KEGG analysis was done. The KEGG output results incorporate KEGG orthology (KO) assignments and corresponding enzyme commission (EC) numbers and metabolic pathways of genes by utilizing “KEGG automated annotation server (KAAS) (http://www.genome.jp/kaas-bin/kaasmain)” [62]. Islandviewer4 was used for the prediction of the genomic islands and antiSMASH 3.0 for secondary metabolite and antimicrobial cluster prediction [63, 64].

## Supplementary information

**Additional file 1: Figure S1.** Genes engaged in respiratory mechanism of *Burkholderia* sp. ISTR5. **Figure S2.** Transcriptional regulators involved in the regulation of various functions in *Burkholderia* sp. ISTR5. **Figure S3.** Transporters responsible for the movement of substrates and ions in *Burkholderia* sp. ISTR5. **Figure S4.** Oxidoreductases responsible aromatic compounds degradation found in *Burkholderia* sp. ISTR5. **Figure S5.** Representation of various stress regulation proteins found in *Burkholderia* sp. ISTR5. **Table S1.** KEGG Pathway classification of R5. **Table S2.** Representation of monooxygenases present in *Burkholderia* sp. ISTR5. **Table S3.** Different dioxygenases found in *Burkholderia* sp. ISTR5. **Table S4.** Peroxidases found in genome of *Burkholderia* sp. ISTR5. **Table S5.** Peripheral pathway for aromatic compounds degradation in *Burkholderia* sp. ISTR5. **Table S6.** Metabolism of central aromatic hydrocarbons in *Burkholderia* sp. ISTR5. **Table S7.** Genes responsible for overcoming stress conditions.

## Data Availability

The additional data generated during this study are available in Additional file 1.

## Abbreviations

LDC: lignin-derived compounds
TOC: total organic carbon
MSM: mineral salt media
LiP: lignin peroxidase
MnP: manganese peroxidase
