# Supplementary material for: Genomic analysis of Burkholderia sp. ISTR5 for biofunneling of lignin-derived compounds
Source: Biotechnol Biofuels. 2019 Nov 27;12:277. doi: 10.1186/s13068-019-1606-5 (PMC6880542; doi:10.1186/s13068-019-1606-5)

Additional file 1

Title: Genomic analysis of ***Burkholderia* sp*.* ISTR5 for biofunneling of Lignin Model Compounds**

Author names and affiliations:
Raj Morya, Madan Kumar, Shashi Shekhar Singh, Indu Shekhar Thakur*

School of Environmental Sciences, Jawaharlal Nehru University, New Delhi- 110067, India

*Corresponding Author

Raj Morya: [rajmr9@gmail.com](mailto:rajmr9@gmail.com)

Madan Kumar: [madankumar9052@gmail.com](mailto:madankumar9052@gmail.com)

Shashi Shekhar Singh: [shekharsbtjnu@gmail.com](mailto:shekharsbtjnu@gmail.com)

Indu Shekhar Thakur: [isthakur@hotmail.com](mailto:isthakur@hotmail.com)

**
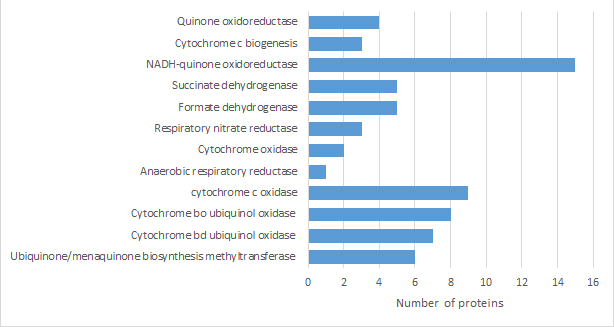
**

Fig. S1. Genes engaged in respiratory mechanism of *Burkholderia* sp. ISTR5


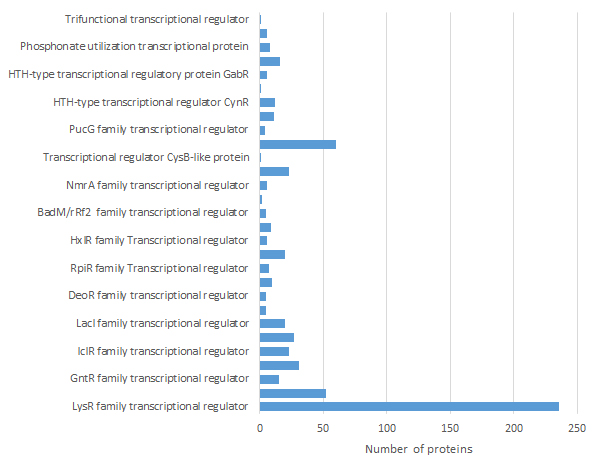


Fig. S2. Transcriptional regulators involved in the regulation of various functions in *Burkholderia* sp. ISTR5


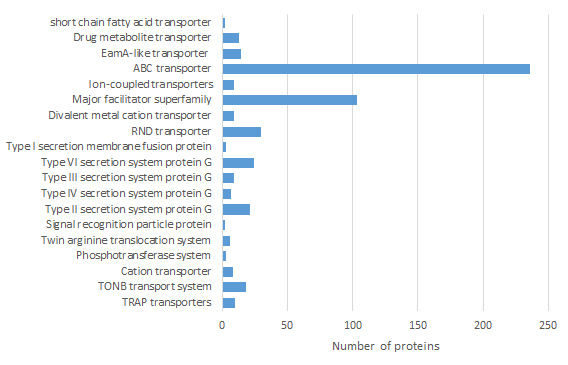


Fig. S3. Transporters responsible for the movement of substrates and ions in *Burkholderia* sp. ISTR5


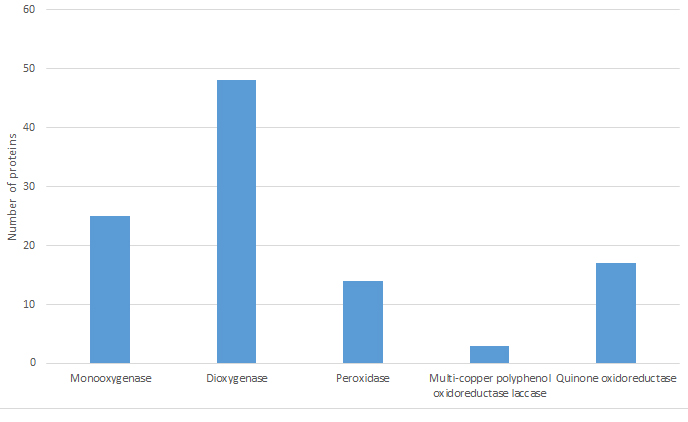


Fig. S4. Oxidoreductases responsible aromatic compounds degradation found in *Burkholderia* sp. ISTR5


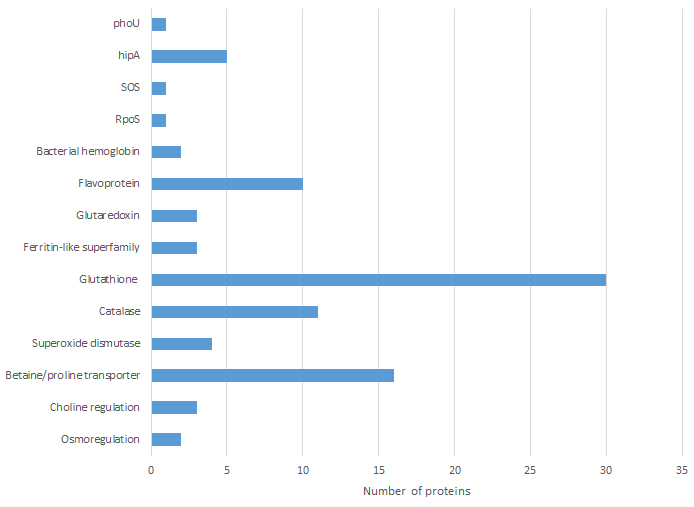


Fig. S5. Representation of various stress regulation proteins found in *Burkholderia* sp. ISTR5


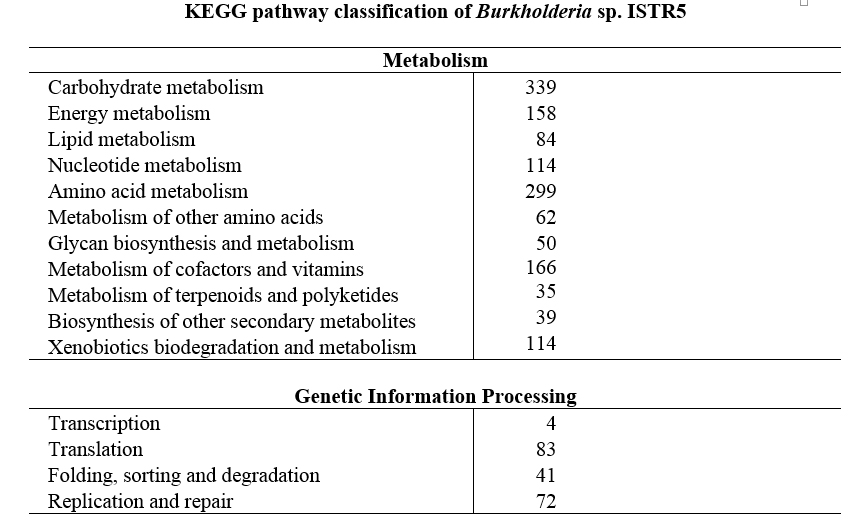
Table S1. KEGG Pathway classification of *Burkholderia* sp. ISTR5


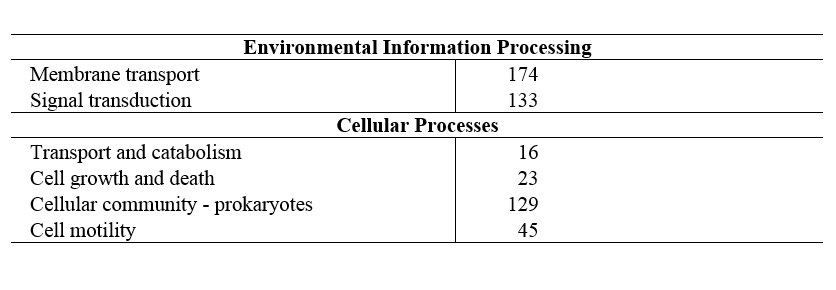


Table S2. Representation of monooxygenases present in *Burkholderia* sp. ISTR5

| Protein | Evalue | Function |
| --- | --- | --- |
| AGENICAA_00356 | 7.30E-209 | Alkanesulfonate monooxygenase |
| AGENICAA_00493 | 9.30E-129 | K03863 vanillate monooxygenase EC 1.14.13.82 |
| AGENICAA_00661 | 1.6e-310 | Baeyer-Villiger monooxygenase |
| AGENICAA_01019 | 4.60E-181 | Putative monooxygenase MoxC |
| AGENICAA_01027 | 7.10E-134 | Nitrilotriacetate monooxygenase component A |
| AGENICAA_01033 | 2.30E-177 | Luciferase-like monooxygenase |
| AGENICAA_01034 | 5.60E-203 | Putative monooxygenase MoxC |
| AGENICAA_01293 | 3.20E-262 | Nitrilotriacetate monooxygenase component A |
| AGENICAA_02163 | 9.00E-154 | Monooxygenase, FAD-binding protein |
| AGENICAA_02278 | 2.60E-292 | Anhydrotetracycline monooxygenase |
| AGENICAA_02346 | 1.00E-260 | Flavin-binding monooxygenase-like |
| AGENICAA_02487 | 6.20E-159 | Phenylacetone monooxygenase |
| AGENICAA_02618 | 3.20E-201 | Alkanesulfonate monooxygenase |
| AGENICAA_02652 | 2.00E-95 | 4-hydroxyphenylacetate 3-monooxygenase reductase |
| AGENICAA_02811 | 9.30E-222 | Putative monooxygenase MoxC |
| AGENICAA_03009 | 1.20E-236 | 4-hydroxybenzoate 3-monooxygenase |
| AGENICAA_03044 | 8.10E-27 | Antibiotic biosynthesis monooxygenase |
| AGENICAA_04049 | 1.20E-172 | Phenylalanine 4-monooxygenase |
| AGENICAA_04321 | 6.20E-227 | 6-hydroxynicotinate 3-monooxygenase |
| AGENICAA_04627 | 1.60E-280 | Dimethyl-sulfide monooxygenase |
| AGENICAA_05661 | 6.60E-199 | Alkanesulfonate monooxygenase |
| AGENICAA_05847 | 3.00E-45 | Antibiotic biosynthesis monooxygenase |
| AGENICAA_06426 | 8.00E-130 | monooxygenase, FAD-binding |
| AGENICAA_06445 | 3.80E-210 | Luciferase-like monooxygenase |
| AGENICAA_06859 | 8.30E-187 | Alkane 1-monooxygenase |

Table S3. Different dioxygenases found in *Burkholderia* sp. ISTR5

| Protein | Evalue | Function |
| --- | --- | --- |
| AGENICAA_00027 | 3.00E-221 | dioxygenase |
| AGENICAA_00490 | 1.30E-64 | Aromatic-ring-hydroxylating dioxygenase beta subunit |
| AGENICAA_00528 | 8.70E-78 | glyoxalase bleomycin resistance protein dioxygenase |
| AGENICAA_00637 | 1.40E-192 | glyoxalase bleomycin resistance protein dioxygenase |
| AGENICAA_00768 | 1.30E-76 | Glyoxalase/Bleomycin resistance protein/Dioxygenase superfamily |
| AGENICAA_00774 | 3.00E-61 | glyoxalase bleomycin resistance protein dioxygenase |
| AGENICAA_00907 | 1.70E-210 | Taurine catabolism dioxygenase TauD, TfdA family |
| AGENICAA_01097 | 1.60E-174 | dioxygenase |
| AGENICAA_01105 | 1.40E-232 | Phytanoyl-CoA dioxygenase |
| AGENICAA_01135 | 2.60E-60 | glyoxalase bleomycin resistance protein dioxygenase |
| AGENICAA_01182 | 2.00E-78 | dioxygenase, subunit beta |
| AGENICAA_01264 | 3.40E-189 | taurine catabolism dioxygenase taud tfda |
| AGENICAA_01298 | 1.80E-147 | Taurine dioxygenase |
| AGENICAA_01319 | 2.20E-97 | Glyoxalase/Bleomycin resistance protein/Dioxygenase superfamily |
| AGENICAA_01397 | 2.80E-67 | glyoxalase bleomycin resistance protein dioxygenase |
| AGENICAA_01503 | 1.70E-174 | Taurine dioxygenase |
| AGENICAA_02081 | 2.70E-175 | 2-Nitropropane dioxygenase |
| AGENICAA_02423 | 4.70E-83 | glyoxalase bleomycin resistance protein dioxygenase |
| AGENICAA_02448 | 1.20E-266 | homogentisate 1,2-dioxygenase (EC 1.13.11.5) |
| AGENICAA_02494 | 2.90E-22 | Gentisate 1,2-dioxygenase |
| AGENICAA_02585 | 4.40E-86 | glyoxalase bleomycin resistance protein dioxygenase |
| AGENICAA_02621 | 2.50E-113 | cysteine dioxygenase type I |
| AGENICAA_02710 | 9.60E-80 | Glyoxalase Bleomycin resistance protein (Dioxygenase |
| AGENICAA_02785 | 3.00E-72 | glyoxalase bleomycin resistance protein dioxygenase |
| AGENICAA_02794 | 6.50E-159 | Phytanoyl-CoA dioxygenase |
| AGENICAA_02822 | 2.90E-171 | Phytanoyl-CoA dioxygenase |
| AGENICAA_02939 | 2.40E-68 | Terephthalate 1,2-dioxygenase oxygenase component |
| AGENICAA_02947 | 9.40E-259 | Protocatechuate 4,5-dioxygenase subunit beta |
| AGENICAA_03294 | 6.20E-76 | glyoxalase bleomycin resistance protein dioxygenase |
| AGENICAA_03356 | 1.20E-65 | glyoxalase bleomycin resistance protein dioxygenase |
| AGENICAA_06948 | 5.60E-217 | 4-hydroxyphenylpyruvate dioxygenase (EC 1.13.11.27) |
| AGENICAA_03861 | 3.10E-145 | Phytanoyl-CoA dioxygenase |
| AGENICAA_03930 | 8.10E-76 | glyoxalase bleomycin resistance protein dioxygenase |
| AGENICAA_04205 | 2.00E-126 | 2-Nitropropane dioxygenase |
| AGENICAA_04300 | 1.70E-57 | Glyoxalase Bleomycin resistance protein (Dioxygenase |
| AGENICAA_04332 | 1.80E-78 | glyoxalase bleomycin resistance protein dioxygenase |
| AGENICAA_04345 | 1.90E-74 | glyoxalase bleomycin resistance protein dioxygenase |
| AGENICAA_04741 | 4.80E-106 | protocatechuate 3,4-dioxygenase subunit alpha |
| AGENICAA_04742 | 2.70E-140 | protocatechuate 3,4-dioxygenase subunit beta |
| AGENICAA_04784 | 2.70E-214 | 4-hydroxyphenylpyruvate dioxygenase (EC 1.13.11.27 |
| AGENICAA_04929 | 8.40E-219 | 2-Nitropropane dioxygenase |
| AGENICAA_05151 | 2.00E-65 | dopa 4,5-dioxygenase |
| AGENICAA_05162 | 4.90E-35 | glyoxalase extradiol ring-cleavage dioxygenase protein |
| AGENICAA_05378 | 4.40E-86 | dioxygenase |
| AGENICAA_05383 | 2.80E-174 | catechol 1,2-dioxygenase |
| AGENICAA_05994 | 8.70E-151 | Extradiol ring-cleavage dioxygenase, class III |
| AGENICAA_06423 | 9.20E-112 | catechol 2,3-dioxygenase (EC 1.13.11.2) |
| AGENICAA_06893 | 2.20E-189 | 2-nitropropane dioxygenase |
|  |  |  |

Table S4. Peroxidases found in genome of *Burkholderia* sp. ISTR5

| Protein | Evalue | Function |
| --- | --- | --- |
| AGENICAA_02725 | 5.16591E-20 | Catalase-related peroxidase |
| AGENICAA_02805 | 4.4122E-22 | Putative non-heme bromoperoxidase BpoC |
| AGENICAA_05934 | 3.70E-266 | Di-heme cytochrome C peroxidase |
| AGENICAA_03263 | 6.92414E-83 | Thiol peroxidase |
| AGENICAA_03740 | 1.37323E-41 | Non-heme chloroperoxidase |
| AGENICAA_04001 | 7.69591E-39 | Catalase-related peroxidase |
| AGENICAA_00252 | 6.10E-120 | Di-heme cytochrome C peroxidase |
| AGENICAA_05245 | 2.54983E-46 | Catalase-related peroxidase |
| AGENICAA_05354 | 1.45603E-74 | putative deferrochelatase/peroxidase YfeX |
| AGENICAA_06521 | 2.7166E-42 | Non-heme chloroperoxidase |
| AGENICAA_05354 | 1.50E-167 | dyp-type peroxidase family protein |
| AGENICAA_05490 | 3.90E-90 | Glutathione peroxidase |
| AGENICAA_04685 | 1.80E-279 | cytochrome C peroxidase |
| AGENICAA_02832 | 3.60E-197 | Dyp-type peroxidase |
|  |  |  |

Table S5. Peripheral pathway for aromatic compounds degradation in *Burkholderia* sp. ISTR5

| Protein | Evalue | Function |
| --- | --- | --- |
| **Salicylate ester degradation** | | |
| AGENICAA_04321 | 2.2674E-52 | 6-hydroxynicotinate 3-monooxygenase |
| AGENICAA_05847 | 3.0732E-17 | Putative monooxygenase YcnE |
| AGENICAA_01034 | 1.289E-63 | Putative monooxygenase MoxC |
|  |  |  |
|  |  |  |
| **Toluene degradation** | | |
| AGENICAA_00628 | 1.2351E-42 | Toluene-4-sulfonate monooxygenase system iron-sulfur subunit TsaM1 |
| AGENICAA_03178 | 1.00E-103 | Toluene tolerance, Ttg2 |
| AGENICAA_06060 | 1.60E-112 | toluene tolerance family protein |
|  |  |  |
|  |  |  |
| **Benzoate degradation** | | |
| AGENICAA_02549 | 8.80E-137 | Major facilitator superfamily MFS_1 |
| AGENICAA_00241 | 9.00E-216 | Major facilitator superfamily |
| AGENICAA_00910 | 8.60E-295 | acyl-CoA dehydrogenase |
| AGENICAA_01720 | 2.20E-217 | Benzoylformate decarboxylase |
| AGENICAA_06180 | 3.80E-206 | benzoate transporter |
|  |  |  |
|  |  |  |
| **p-Hydroxybenzoate degradation** | | |
| AGENICAA_03009 | 1.20E-236 | 4-hydroxybenzoate 3-monooxygenase |
| AGENICAA_05993 | 9.10E-105 | 3-octaprenyl-4-hydroxybenzoate carboxy-lyase |
| AGENICAA_02161 | 1.70E-244 | Major facilitator superfamily, (MFS_1) |
|  |  |  |
|  |  |  |
| **Gentisate degradation** | | |
| AGENICAA_00393 | 7.066E-101 | Ureidoglycolate lyase |
| AGENICAA_06304 | 7.7443E-22 | Glutathione S-transferase |
| AGENICAA_02936 | 6.5182E-09 | 4-hydroxybenzoate transporter PcaK |
| AGENICAA_02448 | 1.20E-266 | homogentisate 1,2-dioxygenase (EC 1.13.11.5) |
| AGENICAA_02494 | 2.90E-22 | Gentisate 1,2-dioxygenase |
| AGENICAA_03301 | 6.50E-102 | ureidoglycolate hydrolase |
| AGENICAA_05197 | 1.90E-96 | Ureidoglycolate hydrolase |
| AGENICAA_05590 | 1.50E-92 | ureidoglycolate hydrolase |
| AGENICAA_02036 | 9.90E-189 | fumarylacetoacetate (faa) hydrolase |
| AGENICAA_04726 | 5.40E-136 | fumarylacetoacetate (faa) hydrolase |
| AGENICAA_05708 | 8.50E-219 | Fumarylacetoacetate hydrolase |
| AGENICAA_06424 | 2.00E-97 | Fumarylacetoacetate (FAA) hydrolase family |
| AGENICAA_01943 | 1.40E-119 | maleylacetoacetate isomerase |
| AGENICAA_02450 | 6.80E-125 | maleylacetoacetate isomerase |
| AGENICAA_02493 | 1.60E-54 | Maleylacetoacetate isomerase |
| AGENICAA_03009 | 1.20E-236 | 4-hydroxybenzoate 3-monooxygenase |
| AGENICAA_00182 | 3.30E-90 | (Mfs) transporter |
| AGENICAA_01323 | 1.90E-227 | Major facilitator superfamily, (MFS_1) |
|  |  |  |
|  |  |  |
| **Chloroaromatic degradation pathway** | | |
| AGENICAA_02686 | 4.2985E-31 | 3-oxoadipate enol-lactonase 2 |
| AGENICAA_03007 | 9.5811E-95 | 3-oxoadipate enol-lactonase 2 |
| AGENICAA_03008 | 1.391E-106 | 3-oxoadipate CoA-transferase subunit A |
|  |  |  |
|  |  |  |
| **Aromatic Amin Catabolism** | | |
| AGENICAA_05950 | 3.40E-78 | phenylacetic acid degradation protein |
| AGENICAA_02652 | 2.00E-95 | 4-hydroxyphenylacetate 3-monooxygenase reductase |
| AGENICAA_00701 | 2.20E-38 | Flavin reductase domain protein, FMN-binding |
| AGENICAA_00457 | 5.70E-209 | NADH flavin oxidoreductase, NADH oxidase |
|  |  |  |
|  |  |  |
| **Lignin degradation fragments** | | |
| AGENICAA_03004 | 5.50E-66 | 4-carboxymuconolactone decarboxylase |
| AGENICAA_00019 | 8.60E-163 | LysR family Transcriptional regulator |
| AGENICAA_00028 | 3.50E-172 | LysR substrate binding domain |
| AGENICAA_02947 | 9.40E-259 | Protocatechuate 4,5-dioxygenase subunit beta |
| AGENICAA_04741 | 4.80E-106 | protocatechuate 3,4-dioxygenase subunit alpha |
| AGENICAA_04742 | 2.70E-140 | protocatechuate 3,4-dioxygenase subunit beta |
|  |  |  |
|  |  |  |
| **Vanillin degradation** | | |
| AGENICAA_00493 | 9.30E-129 | K03863 vanillate monooxygenase EC 1.14.13.82 |
| AGENICAA_04705 | 2.50E-265 | Vanillin dehydrogenase |
| AGENICAA_02886 | 1.60E-298 | Feruloyl esterase |
| AGENICAA_01939 | 3.20E-49 | 4Fe-4S ferredoxin, iron-sulfur binding |
|  |  |  |
| **Ferulate degradation** | | |
| AGENICAA_02886 | 1.60E-298 | Feruloyl esterase |
| AGENICAA_02889 | 7.30E-170 | feruloyl-CoA synthase |
|  |  |  |
|  |  |  |
| **Biphenyl or cinnamic acid degradation** | | |
| AGENICAA_00151 | 6.7956E-92 | 2-ketogluconate reductase |
| AGENICAA_00394 | 1.6533E-48 | 2-keto-3-deoxy-L-fuconate dehydrogenase |
| AGENICAA_00698 | 2.436E-08 | 2-keto-4-pentenoate hydratase |
| AGENICAA_04614 | 2.3196E-52 | L-2-keto-3-deoxyarabonate dehydratase |
| AGENICAA_03129 | 2.8548E-19 | Biphenyl dioxygenase subunit beta |
| AGENICAA_02816 | 6.3988E-40 | 4-hydroxy-2-oxovalerate aldolase |
|  |  |  |
|  |  |  |
| **Phenol degradation** | | |
| AGENICAA_04649 | 1.80E-190 | Cytochrome bd-I ubiquinol oxidase subunit 2 |
| AGENICAA_04950 | 4.70E-147 | Multi-copper polyphenol oxidoreductase laccase |
|  |  |  |
|  |  |  |
| **Phenylacetic acid degradation** | | |
| AGENICAA_02652 | 2.00E-95 | 4-hydroxyphenylacetate 3-monooxygenase reductase |
| AGENICAA_06939 | 3.50E-197 | Phenylacetate-CoA oxygenase subunit PaaA |
| AGENICAA_06940 | 3.40E-49 | phenylacetate-CoA oxygenase subunit paab |
| AGENICAA_06941 | 1.50E-150 | Phenylacetate-CoA oxygenase, PaaI subunit |
| AGENICAA_06942 | 7.00E-102 | Phenylacetate-CoA oxygenase PaaJ subunit |
| AGENICAA_06943 | 2.70E-208 | Phenylacetate-CoA oxygenase reductase subunit PaaK |
| AGENICAA_05638 | 3.90E-123 | Arylesterase (EC 3.1.1.2) |
| AGENICAA_05950 | 3.40E-78 | Acyl-coenzyme A thioesterase PaaI |
|  |  |  |
|  |  |  |
| **Phenylpropionic acid and cinnamic acid degradation** | | |
| AGENICAA_03128 | 7.5164E-44 | 3-phenylpropionate/cinnamic acid dioxygenase subunit alpha |

**Table S6. Metabolism of central aromatic hydrocarbons in *Burkholderia* sp. ISTR5**

| Protein | Evalue | Function |
| --- | --- | --- |
| Catechol branch of beta-ketoadipate pathway | | |
| AGENICAA_05383 | 3.24899E-37 | Catechol 1,2-dioxygenase 2 |
| AGENICAA_01097 | 2.23987E-48 | Hydroxyquinol 1,2-dioxygenase |
| AGENICAA_02178 | 2.11535E-97 | (S)-mandelate dehydrogenase |
| AGENICAA_06726 | 3.2523E-104 | (S)-mandelate dehydrogenase |
| AGENICAA_02686 | 4.29853E-31 | 3-oxoadipate enol-lactonase 2 |
| AGENICAA_03005 | 2.30751E-31 | 3-oxoadipate enol-lactonase 2 |
| AGENICAA_06444 | 2.10207E-32 | 3-oxoadipate enol-lactonase 2 |
| AGENICAA_04267 | 3.38322E-44 | Putative aminoacrylate hydrolase RutD |
| AGENICAA_05864 | 2.47956E-31 | Putative aminoacrylate peracid reductase RutC |
| AGENICAA_06391 | 4.85246E-09 | Putative aminoacrylate hydrolase RutD |
| AGENICAA_03007 | 9.58108E-95 | 3-oxoadipate CoA-transferase subunit B |
| AGENICAA_03008 | 1.3908E-106 | 3-oxoadipate CoA-transferase subunit A |
| AGENICAA_03402 | 1.7932E-147 | 3-methyl-2-oxobutanoate hydroxymethyltransferase |
| AGENICAA_02488 | 1.3592E-120 | putative succinyl-CoA:3-ketoacid coenzyme A transferase subunit A |
| AGENICAA_02489 | 2.4451E-110 | putative succinyl-CoA:3-ketoacid coenzyme A transferase subunit B |
| AGENICAA_05866 | 4.5048E-114 | putative succinyl-CoA:3-ketoacid coenzyme A transferase subunit B |
| AGENICAA_05867 | 1.4913E-117 | putative succinyl-CoA:3-ketoacid coenzyme A transferase subunit A |

| Salicylate and gentisate catabolism | | |
| --- | --- | --- |
| AGENICAA_00922 | 4.6856E-81 | putative FAD-linked oxidoreductase |
| AGENICAA_01878 | 7.0663E-59 | putative FAD-linked oxidoreductase |
| AGENICAA_03381 | 6.0358E-38 | putative FAD-linked oxidoreductase |
| AGENICAA_03382 | 1.559E-123 | putative FAD-linked oxidoreductase |
| AGENICAA_03383 | 2.98E-132 | putative FAD-linked oxidoreductase |
| AGENICAA_05711 | 4.843E-108 | putative FAD-linked oxidoreductase |
| AGENICAA_05879 | 1.161E-127 | putative FAD-linked oxidoreductase |
| AGENICAA_04321 | 2.2674E-52 | 6-hydroxynicotinate 3-monooxygenase |
| AGENICAA_00792 | 4.2743E-11 | 3-hydroxybenzoate transporter MhbT |
| AGENICAA_02234 | 1.7342E-57 | Arylmalonate decarboxylase |
| AGENICAA_02389 | 1.1729E-06 | hypothetical protein |
| AGENICAA_02711 | 5.9268E-59 | Aspartate racemase |
| AGENICAA_02711 | 5.2752E-14 | Aspartate racemase |
| AGENICAA_04322 | 9.4038E-88 | Maleate isomerase |
| AGENICAA_00393 | 7.066E-101 | Ureidoglycolate lyase |
| AGENICAA_02949 | 5.2437E-98 | Ureidoglycolate lyase |
| AGENICAA_03301 | 2.0069E-72 | Ureidoglycolate lyase |
| AGENICAA_05124 | 3.935E-105 | Ureidoglycolate lyase |
| AGENICAA_05590 | 2.5348E-83 | Ureidoglycolate lyase |
| AGENICAA_05658 | 3.8378E-82 | Ureidoglycolate lyase |

| Protocatechuate branch of beta-ketoadipate pathway | | |
| --- | --- | --- |
| AGENICAA_00624 | 3.80E-163 | IclR family transcriptional regulator |
| AGENICAA_02947 | 9.40E-259 | Protocatechuate 4,5-dioxygenase subunit beta |
| AGENICAA_04741 | 4.80E-106 | protocatechuate 3,4-dioxygenase subunit alpha |
| AGENICAA_04742 | 2.70E-140 | protocatechuate 3,4-dioxygenase subunit beta |
| AGENICAA_03006 | 2.30E-244 | 3-carboxy-cis,cis-muconate cycloisomerase |
| AGENICAA_03004 | 5.50E-66 | 4-carboxymuconolactone decarboxylase |
| AGENICAA_03005 | 6.30E-146 | 3-oxoadipate enol-lactonase |
| AGENICAA_02488 | 5.10E-123 | 3-oxoacid CoA-transferase subunit A (EC 2.8.3.5) |
| AGENICAA_01860 | 1.10E-221 | acetyL-CoA acetyltransferase |
| AGENICAA_06596 | 3.50E-217 | acetyl-coa acetyltransferase |
| AGENICAA_04713 | 1.5821E-06 | Alpha-ketoglutarate permease |
| AGENICAA_04858 | 5.60E-82 | Major facilitator superfamily MFS_1 |

| N-heterocyclic aromatic compound degradation | | |
| --- | --- | --- |
| AGENICAA_02421 | 9.60E-114 | isoquinoline 1-oxidoreductase, alpha subunit |
| AGENICAA_00172 | 1.30E-274 | Aldehyde dehydrogenase |
| AGENICAA_00626 | 1.90E-56 | Rieske 2Fe-2S |
| AGENICAA_04853 | 5.40E-186 | Cys/Met metabolism PLP-dependent enzyme |

| **Central meta-cleavage pathway of aromatic compound degradation** | | |
| --- | --- | --- |
| AGENICAA_02947 | 9.40E-259 | Protocatechuate 4,5-dioxygenase subunit beta |
| AGENICAA_00393 | 5.20E-162 | 5-carboxymethyl-2-hydroxymuconate Delta-isomerase |
| AGENICAA_06587 | 6.3436E-12 | 2-hydroxy-6-oxononadienedioate |
| AGENICAA_00261 | 1.40E-40 | 4-oxalocrotonate tautomerase |
| AGENICAA_00788 | 6.338E-127 | 4-hydroxy-2-oxo-heptane-1,7-dioate aldolase |
| AGENICAA_01099 | 2.49E-130 | Phenylacetaldehyde dehydrogenase |

| Homogentisate pathway of aromatic compound degradation | | |
| --- | --- | --- |
| AGENICAA_02448 | 1.20E-266 | homogentisate 1,2-dioxygenase (EC 1.13.11.5) |
| AGENICAA_04784 | 2.70E-214 | 4-hydroxyphenylpyruvate dioxygenase (EC 1.13.11.27 |
| AGENICAA_01943 | 1.40E-119 | maleylacetoacetate isomerase |
| AGENICAA_06818 | 5.40E-72 | Merr family transcriptional regulator |
| AGENICAA_00393 | 5.20E-162 | 5-carboxymethyl-2-hydroxymuconate Delta-isomerase |
| AGENICAA_02036 | 9.90E-189 | fumarylacetoacetate (faa) hydrolase |
| AGENICAA_02449 | 6.60E-257 | fumarylacetoacetase EC 3.7.1.2 |
| AGENICAA_04689 | 9.10E-229 | Aromatic amino acid aminotransferase |
| AGENICAA_02819 | 4.90E-188 | brancheD-chain amino acid aminotransferase |
| AGENICAA_00624 | 3.80E-163 | IclR family transcriptional regulator |
| AGENICAA_00007 | 3.356E-128 | HTH-type transcriptional regulator |
|  |  |  |

**Table S7. Genes responsible for overcoming stress conditions**

| Protein | Evalue | Function |
| --- | --- | --- |
| **Glutathione** | | |
| AGENICAA_00129 | 8.90E-119 | Glutathione S-transferase |
| AGENICAA_00335 | 2.50E-49 | Glutathione S-transferase |
| AGENICAA_00456 | 7.20E-116 | Glutathione S-transferase |
| AGENICAA_00769 | 3.30E-121 | Glutathione S-transferase |
| AGENICAA_00770 | 1.20E-132 | glutathione Stransferase |
| AGENICAA_01051 | 1.80E-49 | Glutathione-dependent formaldehyde-activating |
| AGENICAA_01352 | 2.30E-66 | glutathione-dependent formaldehyde-activating Gfa |
| AGENICAA_01737 | 1.10E-215 | s-(hydroxymethyl)glutathione dehydrogenase |
| AGENICAA_01848 | 2.50E-71 | Lactoylglutathione lyase |
| AGENICAA_01991 | 8.10E-180 | glutathione synthase |
| AGENICAA_02206 | 1.40E-111 | glutathione Stransferase |
| AGENICAA_02569 | 2.30E-21 | Glutathione S-transferase |
| AGENICAA_02586 | 2.00E-92 | Glutathione S-transferase |
| AGENICAA_02773 | 3.40E-138 | glutathione Stransferase |
| AGENICAA_03348 | 2.40E-116 | Glutathione S-transferase |
| AGENICAA_03815 | 8.70E-90 | Glutathione S-transferase |
| AGENICAA_04266 | 2.80E-112 | glutathione Stransferase |
| AGENICAA_04343 | 7.30E-164 | Glutathione S-transferase |
| AGENICAA_04373 | 3.30E-110 | Glutathione S-transferase |
| AGENICAA_05305 | 4.90E-123 | Glutathione S-transferase |
| AGENICAA_05490 | 3.90E-90 | Glutathione peroxidase |
| AGENICAA_05556 | 2.50E-130 | glutathione Stransferase |
| AGENICAA_05692 | 9.30E-133 | Glutathione S-transferase |
| AGENICAA_06561 | 1.70E-115 | Glutathione S-transferase |
| AGENICAA_06705 | 7.70E-121 | Glutathione S-transferase |

| **Superoxide Dismutase** |  |  |
| --- | --- | --- |
| AGENICAA_01574 | 8.5393E-23 | Superoxide dismutase [Cu-Zn] |
| AGENICAA_01684 | 8.091E-110 | Superoxide dismutase [Fe] |
| AGENICAA_04208 | 2.767E-100 | Superoxide dismutase [Mn/Fe] |
| AGENICAA_04353 | 9.2273E-86 | Superoxide dismutase [Fe] |

| **Glutaredoxin** | | |
| --- | --- | --- |
| AGENICAA_01983 | 4.70E-42 | Glutaredoxin 3 |
| AGENICAA_04498 | 6.70E-41 | glutaredoxin 2 |
| AGENICAA_05992 | 1.20E-52 | Glutaredoxin |

| **Thioredoxin** |  |  |
| --- | --- | --- |
| AGENICAA_01708 | 4.20E-156 | Thioredoxin |
| AGENICAA_02877 | 1.60E-217 | Thioredoxin |
| AGENICAA_02955 | 8.60E-57 | Thioredoxin |
| AGENICAA_03638 | 7.40E-181 | thioredoxin reductase |
| AGENICAA_03914 | 1.90E-68 | Thioredoxin |
| AGENICAA_04987 | 5.50E-56 | Thioredoxin |
| AGENICAA_06581 | 4.50E-290 | Thioredoxin |

| **Catalase** | | |
| --- | --- | --- |
| AGENICAA_00846 | 2.80E-166 | Catalase |
| AGENICAA_02725 | 5.60E-206 | Catalase |
| AGENICAA_04001 | 1.80E-212 | Catalase |
| AGENICAA_04367 | 4.10E-292 | Catalase (EC 1.11.1.6) |
| AGENICAA_05245 | 2.90E-205 | Catalase |
| AGENICAA_06723 | 5.60E-300 | Catalase |

Fig. S6 GCMS spectrum of LDCs after 72h and 120 h degradation. a) syringic acid 72h, b) syringic acid 120 h, c) p-coumaric acid 72h, d) p-coumaric acid 120h, e) Ferulic acid 72h, f) Ferulic acid 120 h, g) Benzoic acid 72h, h) Benzoic acid 120h


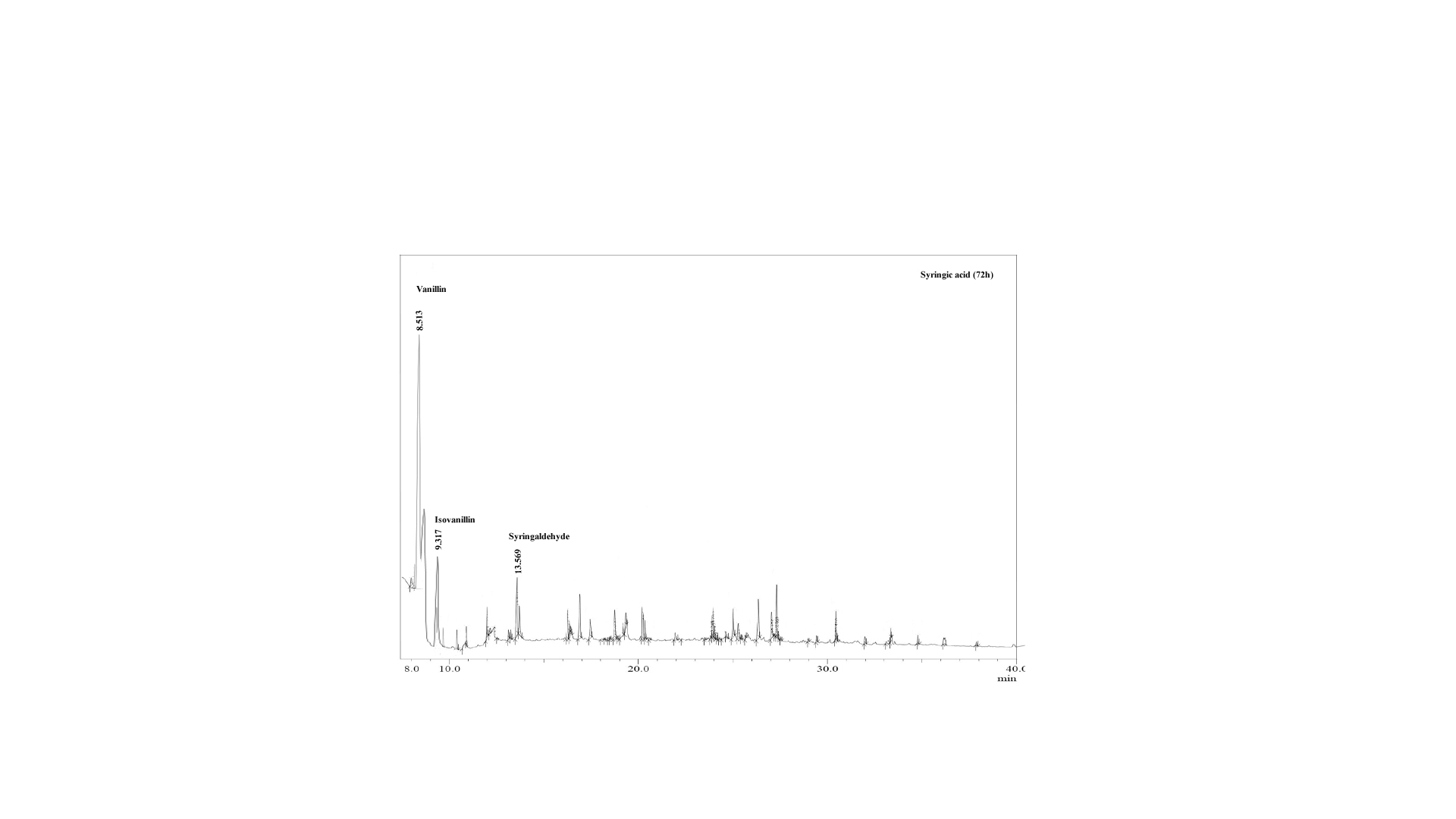


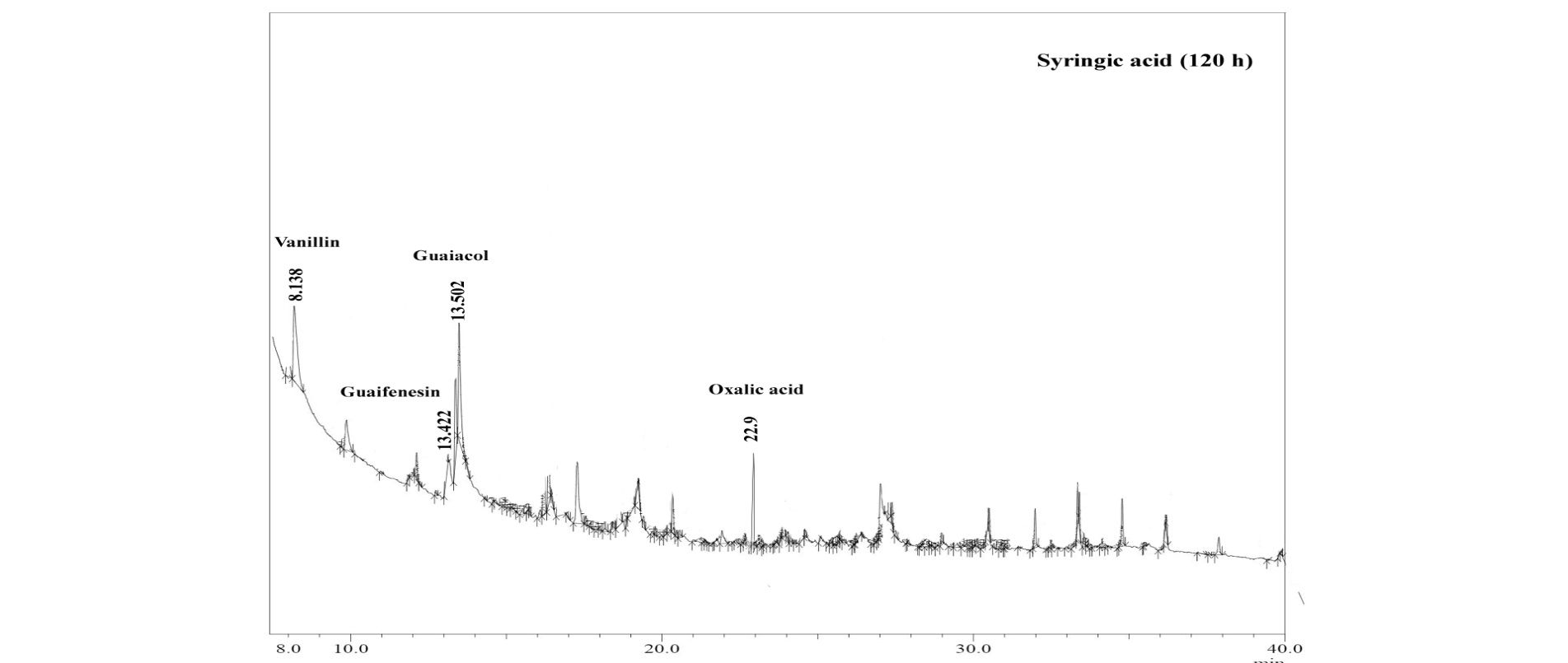


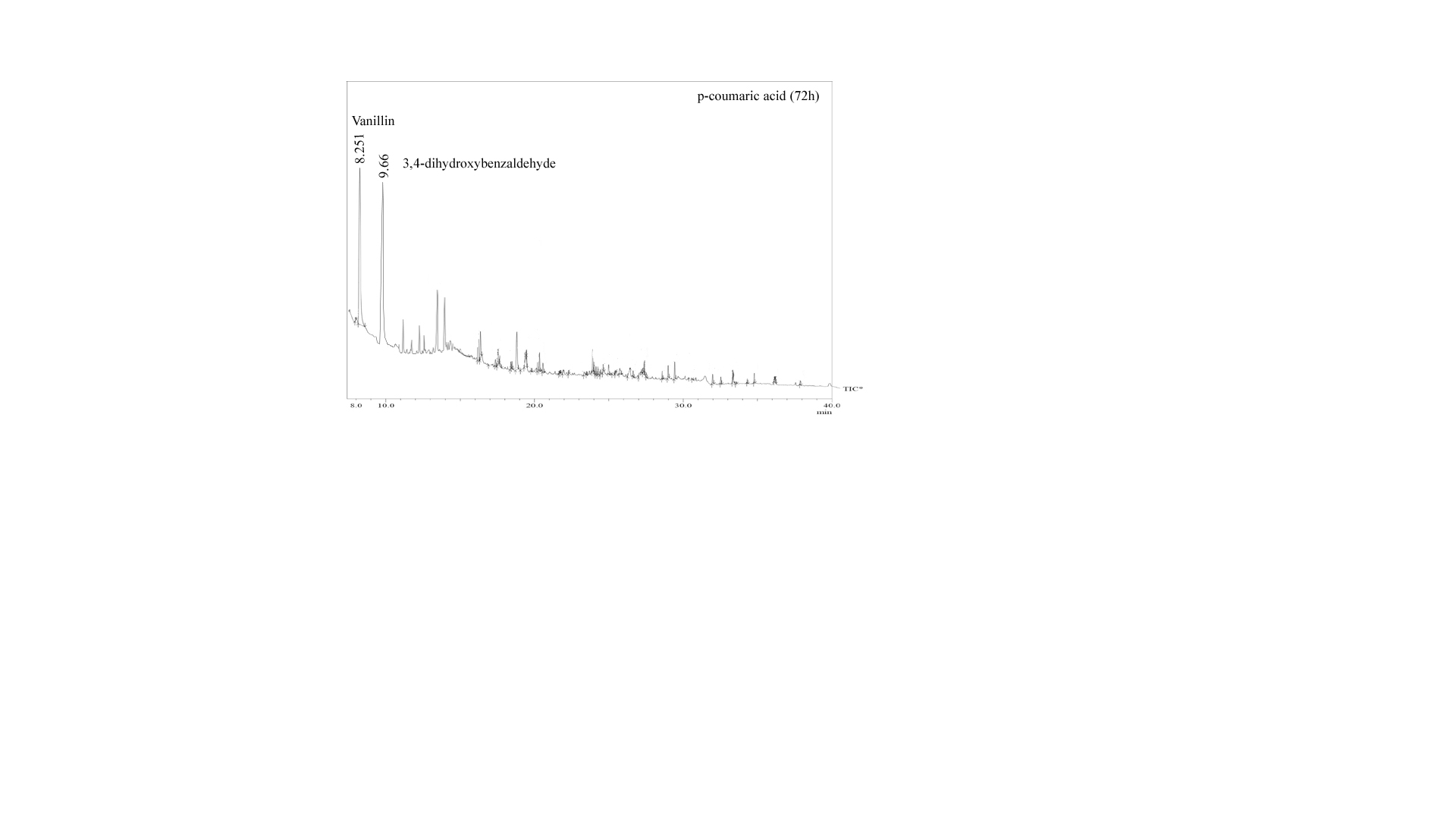


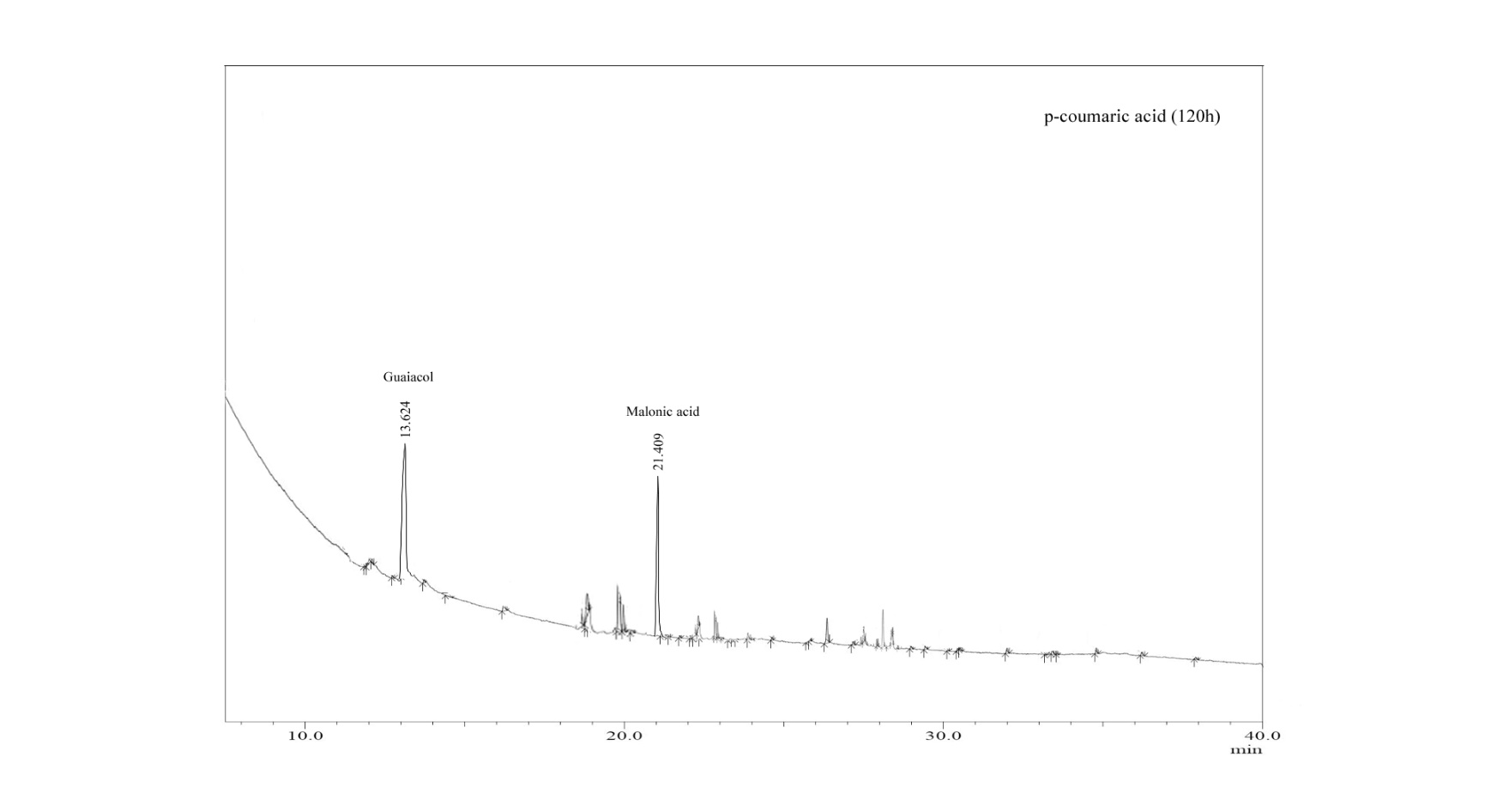


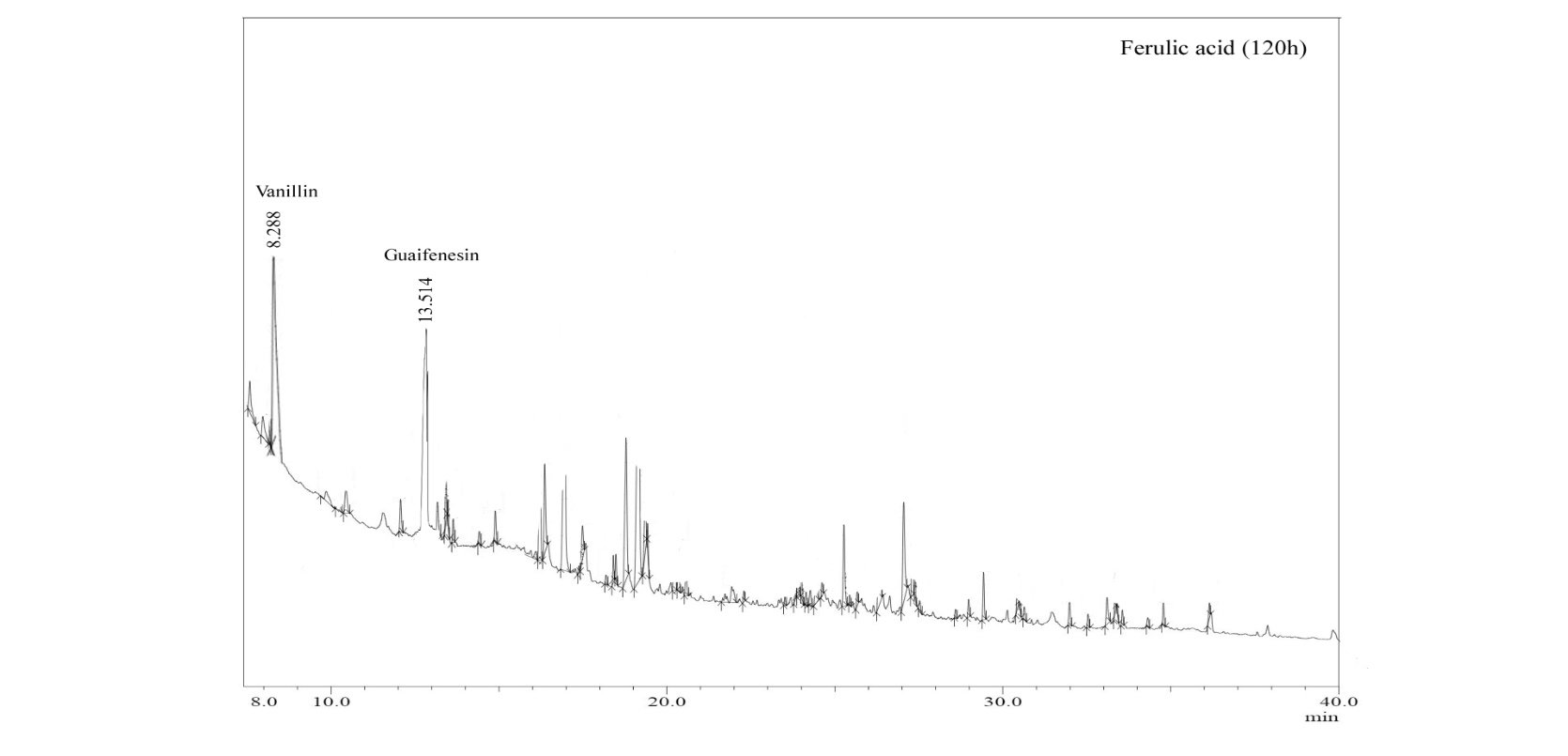

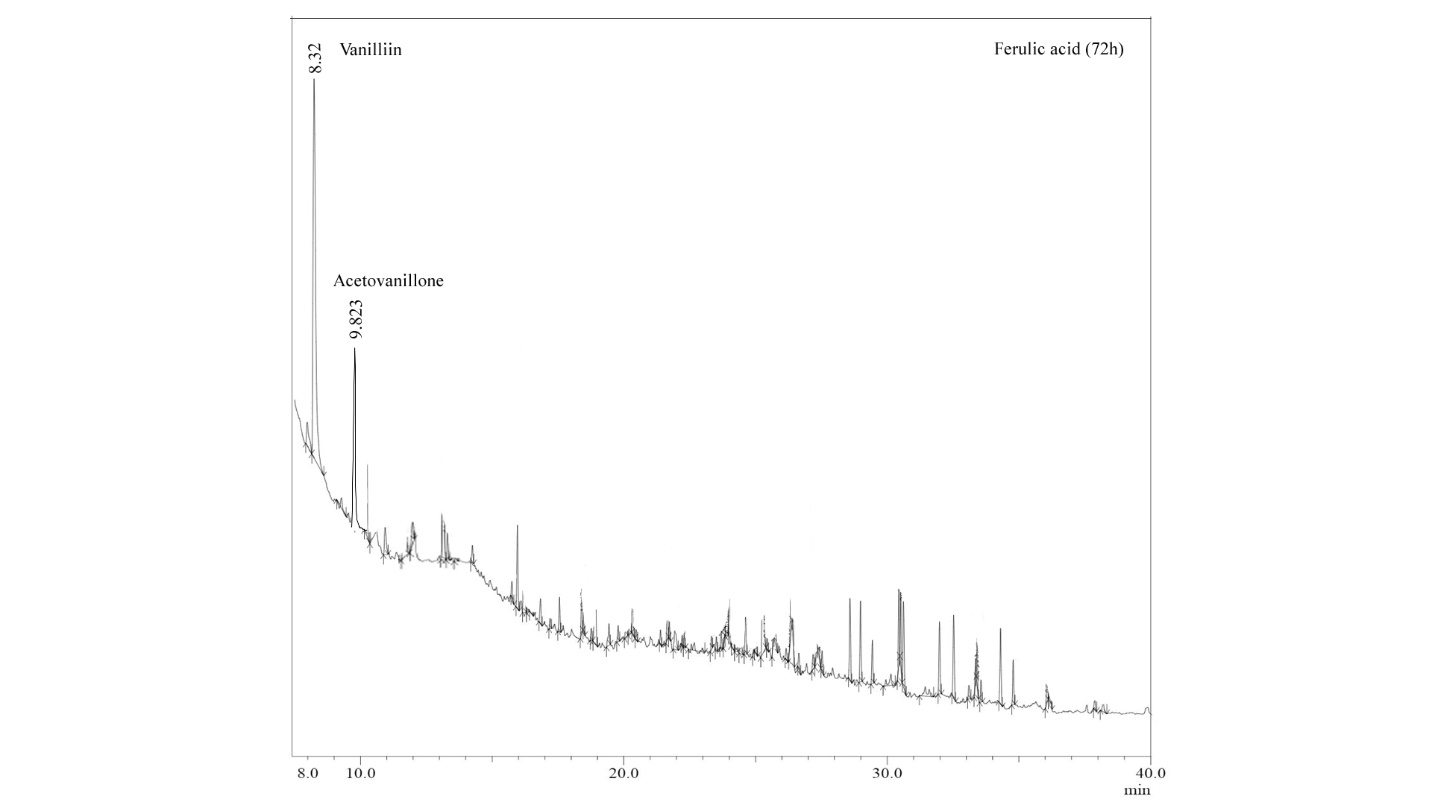


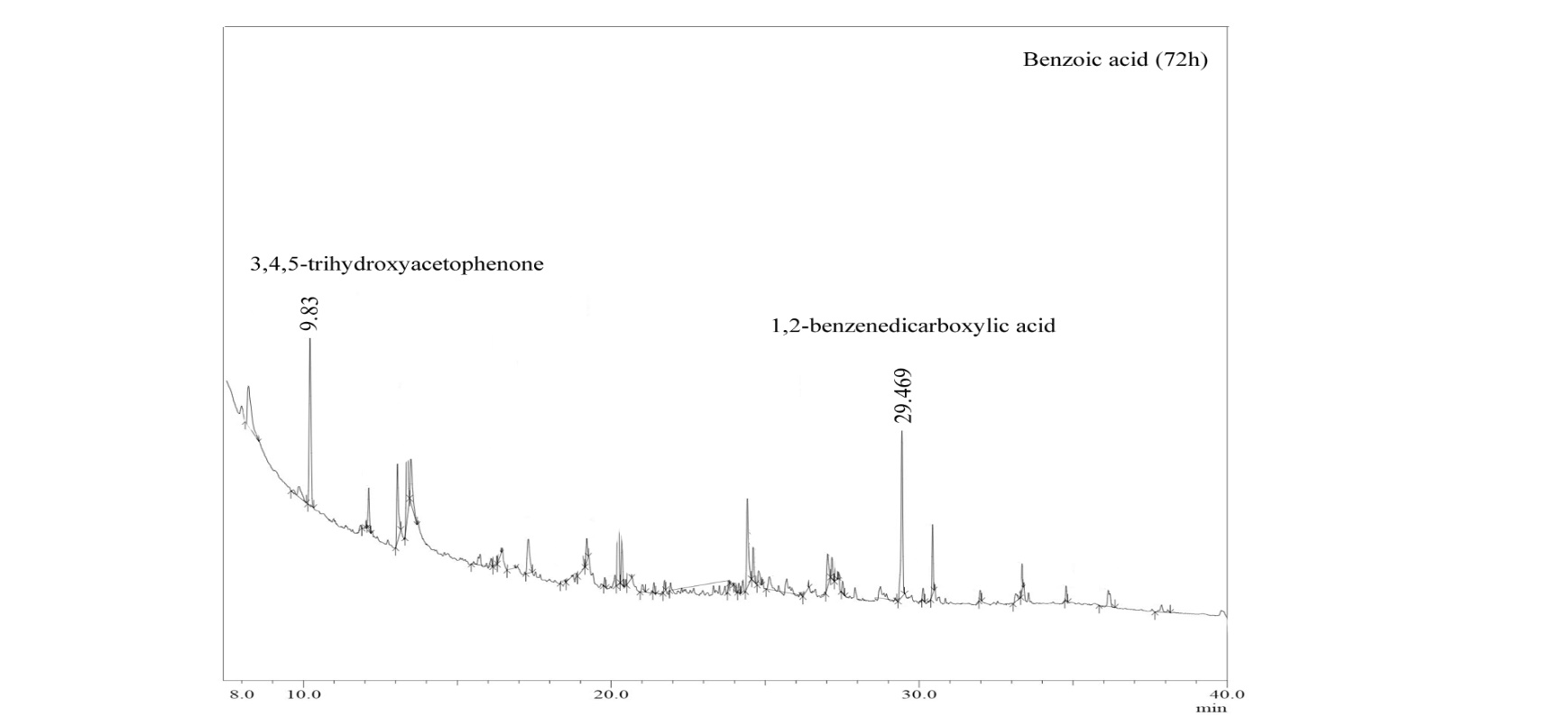


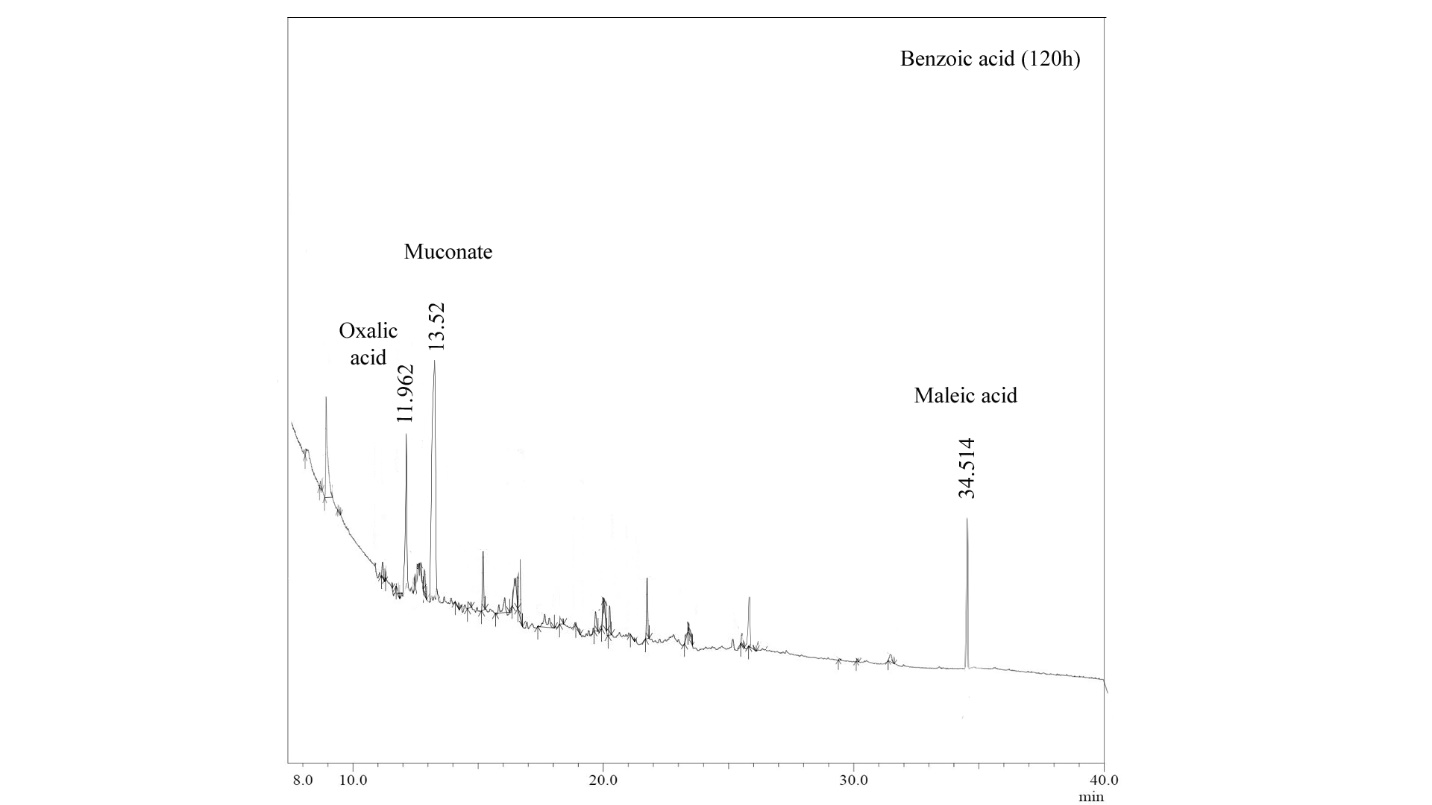

Supplement: Supplementary file 1 — Additional file 1: Figure S1. Genes engaged in respiratory mechanism of Burkholderia sp. ISTR5. Figure S2. Transcriptional regulators involved in the regulation of various functions in Burkholderia sp. ISTR5. Figure S3. Transporters responsible for the movement of substrates and ions in Burkholderia sp. ISTR5. Figure S4. Oxidoreductases responsible aromatic compounds degradation found in Burkholderia sp. ISTR5. Figure S5. Representation of various stress regulation proteins found in Burkholderia sp. ISTR5. Table S1. KEGG Pathway classification of R5. Table S2. Representation of monooxygenases present in Burkholderia sp. ISTR5. Table S3. Different dioxygenases found in Burkholderia sp. ISTR5. Table S4. Peroxidases found in genome of Burkholderia sp. ISTR5. Table S5. Peripheral pathway for aromatic compounds degradation in Burkholderia sp. ISTR5. Table S6. Metabolism of central aromatic hydrocarbons in Burkholderia sp. ISTR5. Table S7. Genes responsible for overcoming stress conditions. [file 13068_2019_1606_MOESM1_ESM.docx]
